# *N*‑Heterocyclic Carbene-Based Group 4 Catalysts for the Terpolymerization of Cyclohexene Oxide and Cyclic Anhydrides with CO_2_

**DOI:** 10.1021/acsorginorgau.5c00002

**Published:** 2025-05-13

**Authors:** Lakshmi Suresh, Kathrin Zwettler, Karl W. Törnroos, William Le, Benoît Marcolini, Gilles Frache, Erwan Le Roux

**Affiliations:** † Department of Chemistry, 1658 University of Bergen, Allégaten 41, N-5007, Bergen, Norway; ‡ 87145 Luxembourg Institute of Science and Technology (LIST), 41, rue du Brill, L-4422, Belvaux, Luxembourg; § Luxembourg Institute of Science and Technology (LIST), 5, avenue des Hauts-Fournaux, L-4362, Esch-sur-Alzette, Luxembourg

**Keywords:** Group 4 Catalysts, N-Heterocyclic Carbene, Cyclohexene Oxide, Cyclic Anhydrides, Carbon Dioxide, Terpolymerization, Copolymerization

## Abstract

A series of bis-phenolate saturated *N*-heterocyclic carbene (NHC) group 4 complexes ([κ^3^-O,C,O]-NHC)­M­(O*i*Pr)­Cl­(THF) (M = Ti, **1**; Zr, **2**; Hf, **3**) in the presence of [PPN]Cl as cocatalyst were investigated and showed high activity in the tandem terpolymerization of phthalic anhydride (PA), cyclohexene oxide (CHO) with CO_2_. The resultant terpolymers revealed a diblock pattern leading selectively to poly­(ester-*b*-carbonate). Subsequently, other titanium complexes ([κ^3^-O,C,O]-NHC)­TiX_2_ bearing various coligands (X = Cl, **4**; O*i*Pr, **5**; OAc, **6**; OAc^F^, **7**) also displayed high activity with a turnover frequency (TOF) up to 460 h^–1^ that is comparable to **1**. Using the same tandem approach, the nature of terpolymers was modulated with other mono- and tricyclic anhydrides alongside CHO with CO_2_. Intrigued by the high rates of PA conversion observed experimentally in terpolymerization, complexes **1**–**3** as well as benzannulated and unsaturated NHC analogues of complex **1** were investigated as a stand-alone reaction for the copolymerization of PA and CHO. Complex **1**/[PPN]Cl displayed excellent catalytic activity (TOF ∼ 1600 h^–1^) and high selectivity (≥99%) toward polyesters comparable to other highly active heteronuclear (Al/K and Fe/K) catalysts and binary (salen)­MX systems. Kinetic studies performed on complexes **1** and **3** determined activation barriers (*E*_a_) consistent with the observed catalytic trend, *i.e*., *E*_a_: Ti < Hf.

## Graphical abstract

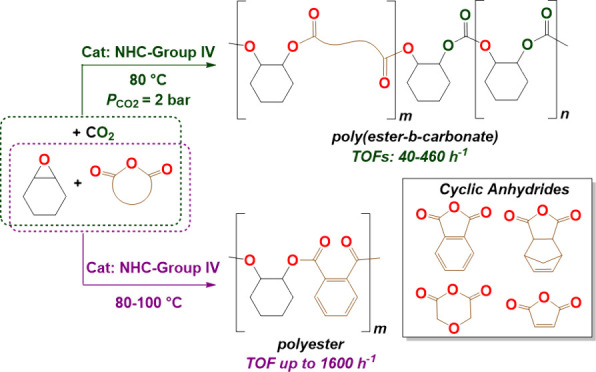

## Introduction

Epoxide and CO_2_ ring-opening copolymerization (ROCOP) enabled by homogeneous metal catalysts have gained widespread attention especially because of its ability to convert CO_2_, a concerning greenhouse gas, into aliphatic polycarbonates.^1–8^ However, the material properties of these sustainable polymers do not often meet the commercial demands.^9,10^ Integrating epoxides of varying structural features into the polymer backbone often shows little improvement in the overall polymer properties. For example, poly­(cyclohexene-*alt*-carbonate) (PCHC) displays better thermal properties compared to poly­(propylene carbonate) (PPC) but suffers from low degradability.^11^ One strategic approach to overcome this challenge is to copolymerize two or more subunits with specific properties into a single polymer chain. By regulation of the composition and sequence distribution of constituent blocks, tailored polymer structures with enriched material properties can be achieved. Particularly in this interest, block polymers consisting of ester and carbonate fragments are highly promising as biodegradable implants.^12,13^ A straightforward method for producing polyesters is the ROCOP of epoxides and cyclic anhydrides.^13–15^ On a mechanistic level, this reaction resembles ROCOP of epoxide and CO_2_ due to a metal-alkoxide propagating unit. Therefore, many metal catalysts previously reported for ROCOP of epoxides with CO_2_ also promote terpolymerization, affording polymers containing ester and carbonate fragments showing enhanced polymer properties.^12,13,16–20^ Moreover, a broad range of cyclic anhydrides are available commercially that allow easy modification of the polymer functionalities.^13–15^ A common outcome of the terpolymerization of cyclic anhydrides, epoxides and CO_2_ is a diblock consisting of polyester and polycarbonate fragment connected through ester-*co*-carbonate junction. Conventionally a diblock terpolymer of this type is represented as poly­(ester-*b*-carbonate).

Among the first highly active homogeneous catalysts reported for the terpolymerization, Coates et al. developed a discrete homogeneous zinc catalyst bearing β-diiminate (BDI) ligand that was able to efficiently polymerize CHO, diglyconic anhydride (DGA) with CO_2_ forming selectively poly­(ester-*b*-carbonate) terpolymer without any cocatalyst (Scheme 1).^21^ Other catalytic systems such as di-Mg and di-Zn based on Robson’s macrocyclic ligands, which does not require a cocatalyst, were investigated by Williams et al. for the terpolymerization of cyclohexene oxide (CHO) with tricyclic anhydride or phthalic anhydride (PA) under a low pressure of CO_2_ (1 bar), yielding in the formation of well-defined blocks of poly­(ester-*b*-carbonate)­s with less than 5% of cyclohexene carbonates (CHCs).^22^ Since these discoveries, other catalysts such as *o*-vanillin and heteroscorpionate based di-Zn,^23,24^ as well as dinuclear Cr salen^25^ and Al^26^ complexes in the presence of neutral 4-dimethylaminopyridine (DMAP) or anionic ([PPN]­Cl) cocatalysts produced selectively diblock terpolymers in the presence of CHO/PA and CO_2_. Like bimetallic systems, monometallic catalysts have displayed broad cyclic anhydride scope, producing various anhydride modulated diblocks. [PPN]­Cl-Activated chromium catalysts based on salen,^27–29^ diamino-bis­(phenolate)^30^ and porphyrin ligands^29^ have been successfully employed for forming diblock terpolymers with succinic anhydride (SA), cyclopentane-1,2-dicarboxylic acid anhydride (CPA) and cyclopropane-1,2-dicarboxylic acid anhydride (CPrA) in addition to PA along with CHO and CO_2_ (Scheme 1).

**Scheme 1:**
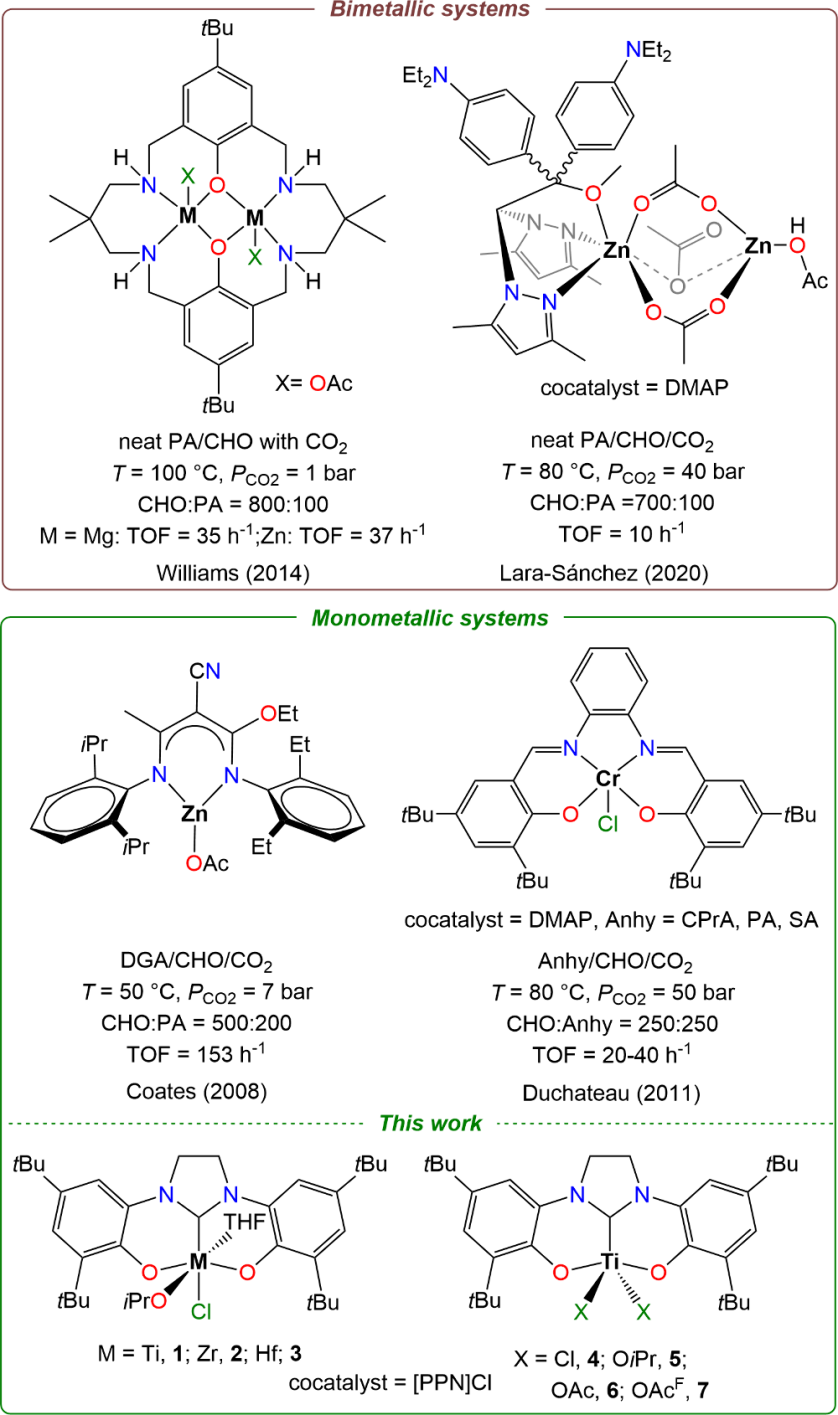
Selected Examples of Catalysts Forming Poly­(ester-*b*-carbonates) by Tandem Terpolymerization

Despite a well-established ROCOP pathway for polyesters and polycarbonates, group 4 metal catalysts in terpolymerization are unexplored.^13,15,17,18^ This is rather surprising considering its prominence in catalyzing epoxide/CO_2_ copolymerization that also seems to proceed via an alkoxide initiating group.^1–8,14,15^ Therefore, we envisaged to explore potential group 4-NHC catalysts, previously known to promote CHO/CO_2_ copolymerization^31–33^ as well as ring-opening polymerization of cyclic esters^34^ in terpolymerization of CHO, PA and CO_2_. A detailed study aimed at catalyst/cocatalyst screening, monomers’ feed ratio, and scope have been investigated. To further support the catalytic trend observed in the terpolymerization, kinetic studies of ROCOP involving CHO and PA were conducted.

## Results and Discussion

### Terpolymerization of CHO, PA and CO_2_

Complexes **1**–**6** ^32,35,36^ were synthesized according to known literature procedures (Scheme 1), and complex **7** was obtained in high yield via salt metathesis reaction of ({κ^3^-O,C,O}-NHC)­Ti­(Cl)_2_ **4** and sodium fluoroacetate (NaOAc^F^) in dichloromethane at room temperature (Figure 1). Complex **7** was characterized by using ^1^H, ^13^C and ^19^F NMR, DRIFT spectroscopy, elemental analysis and single crystal X-ray diffraction (see Supporting Information, Figure 1, Figures S1–S4 and Table S1).

**Figure 1:**
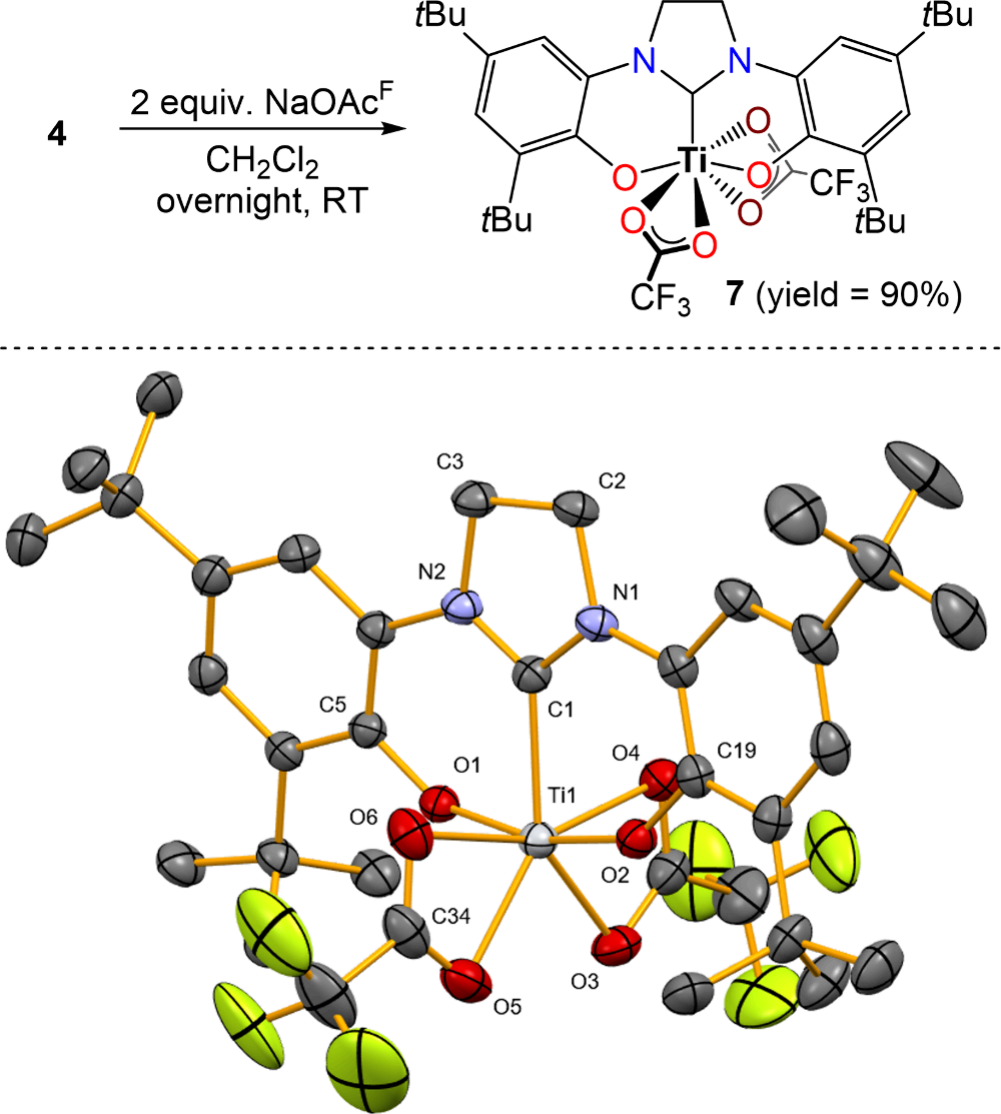
Synthesis of complex **7** and its molecular structure obtained by single crystal XRD with thermal ellipsoids (anisotropic displacement ellipsoids are shown at 50% probability). H atoms are omitted for clarity.

First, complex **1** combined with the cocatalyst [PPN]­Cl was investigated for catalytic activity in the terpolymerization of neat CHO and PA with CO_2_ (2 bar) at 80 °C (Table 1, entry 1). The results indicated full conversion of PA to polyesters (PE) via ROCOP of CHO and PA as well as formation of polycarbonates (PCHC) via ROCOP of CHO and CO_2_ after 45 min of reaction time. When the catalyst loading is decreased by 10-fold without changing other reaction parameters, only polyesters were observed even after 24 h. To assess the reaction progress and the pattern of polymer formation, polymerization was monitored systematically via ^1^H NMR spectroscopy. The ^1^H NMR spectrum acquired after 5 min of reaction showed a proton signal at δ 5.15 ppm pertaining to the methine – (C*H*_*b*_)- protons of polyester (Figure 2A). Additionally, the aromatic protons C*H*_*a*_/C*H*_*b*_ of the polyester chain appeared between δ 7.35 and 7.55 ppm along with free PA (δ 7.85 and 7.95 ppm), accounting for a total of 50% PA conversion. The extremely fast ring-opening of PA also showed the absence of any induction period. On increasing the reaction time to 10 min, proton signals arising from free PA disappeared completely indicating full conversion of PA. It was notable that at this point, the proton signals for PCHC were still absent implying that the PE formation occur prior to the PCHC. When all the PA is fully consumed, CO_2_ insertion is observed into the polymer chain, and results in forming PA-CHO–CO_2_ linkage (C*H*_*d/d’*_) which appeared as two weak and broad signals at δ 4.75 and 5.15 ppm (Figure S5). After a reaction time of 30 min, distinct proton signals corresponding to PCHC appeared at δ 4.65 ppm. With prolonged reaction time, the signal intensity for PCHC increased, confirming polymer growth (Figures 1A and S5). The reaction was quenched after 45 min, and the isolated polymer shows characteristic signals of poly­(ester-*b*-carbonate) as determined by ^1^H NMR spectroscopy (Figure S6). The ^13^C NMR spectrum of the isolated polymer displayed a single carboxylate resonance signal at δ 166.8 ppm corresponding to the ester group of the PE fragment, whereas the multiple signals centered at δ 153.6 ppm indicate the irregular stereochemistry of the carbonate group of the atactic PCHC block (Figure S7). Additionally, diffusion-ordered spectroscopy (DOSY) NMR recording of a single logarithm of the diffusion coefficient (logD) (Figure 2B) indicates that the polyester/carbonate fragments belong to the same polymer chain. These results confirm a diblock poly­(ester-*b*-carbonate) terpolymer.

**Table 1:**
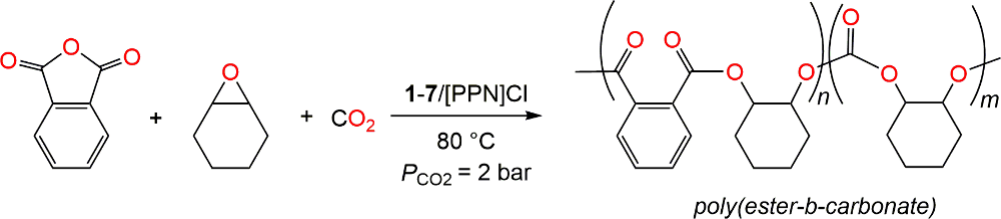
Terpolymerization of CHO, PA and CO_2_ Catalyzed by Complexes **1**–**7** ^a^

Run	Complex	η_PA_ ^b^ (%)	η_CHO_ ^b^ (%)	Sel_PE/PCHC_ ^b^ (%)	TOF (h^–1^)	*M*_n_^c^ (kg mol^–1^)	*Đ* ^c^
1	**1**	100	40	36/64	429	8.6	1.4
2	**2**	100	17	65/35	177	7.1	1.2
3	**3**	100	25	47/53	271	5.9	1.1
4	**4**	100	39	38/62	419	7.9	1.4
5	**5**	100	43	35/65	461	8.0	1.5
6	**6**	100	40	40/60	423	10.6	1.1
7	**7**	100	43	36/64	456	6.5	1.3

a Terpolymerization conditions: complex:[PPN]­Cl:CHO:PA = 1:1:800:100, *P*_CO_2__ = 2 bar, at 80 °C for 45 min.

b Conversion and selectivity determined by ^1^H NMR spectroscopy of the crude mixture.

c Determined by GPC-SEC in THF at 30 °C against polystyrene standard.

**Figure 2:**
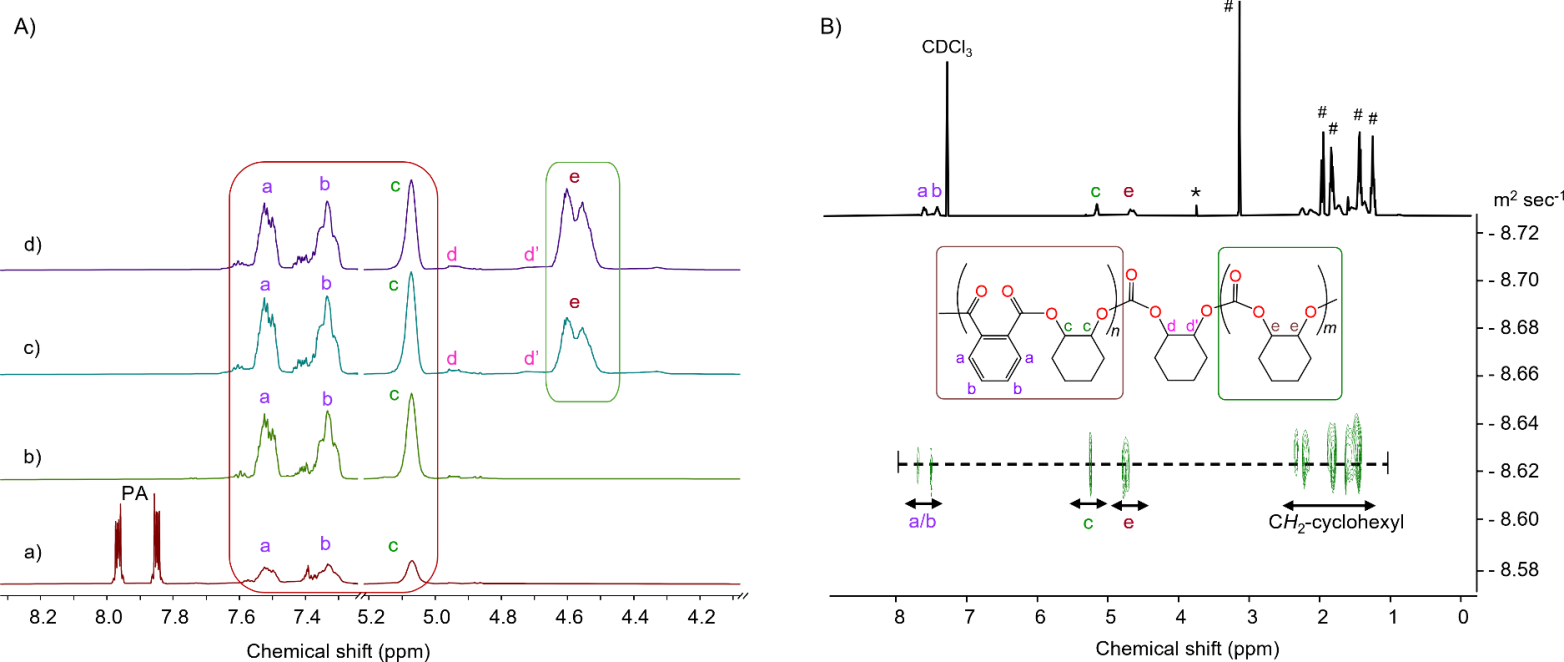
A) Monitoring of the terpolymerization of PA, CHO with CO_2_ in the presence of complex **1**/[PPN]Cl using ^1^H NMR spectroscopy (298 K, 600.13 MHz, chloroform-*d*) after: a) 5 min, b) 10 min, c) 30 min and d) 45 min. B) Full DOSY NMR spectrum (298 K, 600.13 MHz, chloroform-*d*) of poly­(ester-*b*-carbonate) (# = CHO, * = impurity, Table 1, entry 1).

Under similar reaction conditions, both zirconium **2** and hafnium **3** analogues of complex **1** also reported full conversion of PA to polyester (Table 1, entries 2–3) leading to the formation of poly­(ester-*b*-carbonate) terpolymers. Comparative evaluation of their reaction rates revealed superior activity for titanium **1** over other group 4 metals (**3**: TOF = 271 h^–1^ and **2**: TOF = 177 h^–1^). Owing to its superior activity, further terpolymerization studies were performed using complex **1**. Other titanium complexes **4**–**7** bearing various coligands, coupled with [PPN]­Cl, were also tested in terpolymerization. As observed for **1**, all complexes displayed high activity (TOF up 461 h^–1^) with full PA conversion and polymer selectivity (≥99%) for diblock terpolymers (Table 1, entries 4–7) indicating that the coligands seem to be rather insignificant in the overall activity of these complexes.

The GPC-SEC of all the terpolymers obtained displayed a bimodal distribution of low molecular weights (Figure S8) and narrow dispersity indicating controlled polymerization (*Đ* < 1.5). The bimodal behavior is most likely due to the presence of chain transfer agents (CTAs) such as residual moisture and cyclohexane-1,2-diol arising from the hydrolyzed CHO, that give rise to low molecular weight polymers as previously reported.^37–42^

Next, the influence of various cocatalysts on the reaction progress was monitored. Complex **1** without cocatalyst attained 43% of PA conversion to polyester at 80 °C after 45 min of reaction time (TOF 69 h^–1^, Table S2, entry 1–2). This rather slow reaction is also accompanied by concomitant formation of polyether (85%). Anionic nucleophile cocatalysts like [*n*Bu_4_N]Br and [*n*Bu_4_N]Cl as well as neutral cocatalysts such as DMAP and PPh_3_, combined with complex **1** actively produced polyester without proceeding toward polycarbonates (Table S2, entries 4–7). Except for complex **1**/PPh_3_ which converts only 24% PA (Table S2, entry 7), all these cocatalysts in combination with complex **1** showed full PA conversion after 45 min. Unlike [*n*Bu_4_N]­X salts, [PPN]Cl along with **1** was highly active in terpolymerization (Table S2, entry 1). The slower rate of PA conversion (70%) by [PPN]Cl alone when tested in catalysis (Table S2, entry 3) further emphasize the established synergy between cocatalyst and the metal complex during ROCOP catalysis as previously reported for CHO/CO_2_ copolymerization.^43,44^ This is most likely due to the formation of putative titanium “ate” species upon adding [PPN]Cl to titanium complex **1** which enable CO_2_ insertion leading to polycarbonates as previously demonstrated.^44^

The ester:carbonate ratio within a diblock terpolymer may be regulated by adjusting the monomer composition. This is particularly relevant to achieve required thermal properties for the subsequent terpolymer.^45^ Therefore, to control the microstructure of the terpolymers, the monomer feeding ratios were varied. The amounts of PA and CHO were adapted, keeping other reaction parameters constant. At a constant catalyst concentration, for high PA loading, full conversion of cyclic anhydrides to polyester was attained after 45 min. The proton signals pertaining to polycarbonates were absent at this stage (Table 2, entry 1). Under similar conditions, at a lower PA concentration, the reaction proceeded toward polycarbonates following the complete formation of polyesters (Table 2, entry 2). At a constant PA concentration with respect to catalyst, varying the CHO composition still favored polyester formation over polycarbonates (Table 2, entries 3–5). Even at competing monomeric ratios, the catalyst is still selective toward polyester, with 60% conversion of PA within 15 min (Table 2, entry 3). However, the reaction had to be terminated before full conversion of PA due to high viscosity of the reaction mixture that hinders further stirring inside the reactor. These results reveal two key points: *i*) the length of the polyester chain may be regulated by the amount of PA fed into the reaction mixture and *ii*) polyester formation is always favored over polycarbonates indicating the absence of tapering.

**Table 2:** Effect of Monomer Feed Ratio on Terpolymerization of PA, CHO with CO_2_ ^a^

Run^a^	Ratio **1**:[PPN]Cl:CHO:PA	η_PA_ ^b^ (%)	η_CHO_ ^b^ (%)	Sel_PE/PCHC_ ^b^ (%)
1	1:1:1000:200	100	24	99/1
2	1:1:1000:50	100	21	74/26
3^c^	1:1:200:100	60	45	99/1
4	1:1:600:100	100	36	57/43
5	1:1:800:100	100	40	36/64
6^d^	1:1:800:100	100	18	88/12

a Terpolymerization conditions: *P*_CO_2__ = 2 bar at 80 °C for 45 min.

b Conversion and selectivity determined by ^1^H NMR spectroscopy of crude mixture.

c 15 min.

d 100 °C for 10 min.

The thermal analyses of these resulting terpolymers containing various amounts of PE/PCHC content (74/26, 57/43 and 36/64, respectively) along with PE and PCHC alone (Figure S9 and Table S3), revealed single glass transition temperatures (*T*_g_) ranging from 108 to 121 °C indicating that these poly­(ester-*b*-carbonate)­s are miscible (Table S3, entries 2–4). These values are lower than PE (*T*_g_ = 120 °C) with a low content of PCHC (99/1), especially the one with comparable molecular weight (Table S3, entries 1 and 3), showing that the *T*_g_ in the poly­(ester-*b*-carbonate)­s is dominating mostly by the PE content. Nevertheless, for identical PE segments, *i.e*. formed upon full conversion of 100 equiv of PA, the *T*_g_ of poly­(ester-*b*-carbonate)­s can be raised from 107 to 121 °C by slightly increasing the PCHC content as shown in Table S3 (entries 3 and 4). Meaning that *T*_g_’s modulation in these diblock PE–PCHC terpolymers can be further tuned by increasing the PCHC content, as corroborated by the marked *T*_g_ rise from 73 to 118 °C associated with the production of higher molecular weights of PCHC (from *M*_n_ = 8.2 to 21 kg mol^–1^, respectively, Table S3, entries 5–6). The decomposition temperature (*T*_d_) of the poly­(ester-*b*-carbonate)­s determined by thermogravimetric analysis (Figure S10 and Table S3), indicates uniform degradation behavior and the trends observed for the *T*_g_ are similar for the *T*_d_, where increased PE content and longer PCHC chain lengths result in an enhancement of the *T*_d_ (Table S3, entries 1–2 and entries 3–4, respectively). The *T*_g_ and *T*_d_ values found for the poly­(ester-*b*-carbonate)­s are in the expected range of temperatures.^17,22,27,46,47^

Terpolymerization occurs efficiently up to 100 °C (Table 2, entry 6). A further increase in temperature inhibits formation of polycarbonate leading to large amounts of cyclic carbonates in addition to polyester in the reaction medium.^48–53^

### Monomer Scope

Most reports on diblock terpolymers are based on PA/CHO with CO_2_,^22–25^ and therefore, we decided to explore other types of cyclic anhydrides in terpolymerization in the presence of CHO and CO_2_ catalyzed by **1**/[PPN]­Cl (Scheme 2). Monocyclic diglyconic anhydride (DGA) and maleic anhydride (MA) as well as tricyclic norbornene anhydride (NA) were fully consumed according to the ^1^H NMR spectroscopy (Figures S11–13) into corresponding poly­(ester-*b*-carbonate)­s within 4–5 h (Table S4, entries 1–3). All the GPC-SEC analyses of the diblock polymers revealed low molecular weights and narrow polydispersities (Figure S14). Although the rate of polyester formation in all of the above cases is much lower than that for bicyclic phthalic anhydride, the diverse range of polyesters that can be attained using the catalyst is highly beneficial for generating PE modulated polymer structures.

**Scheme 2:**
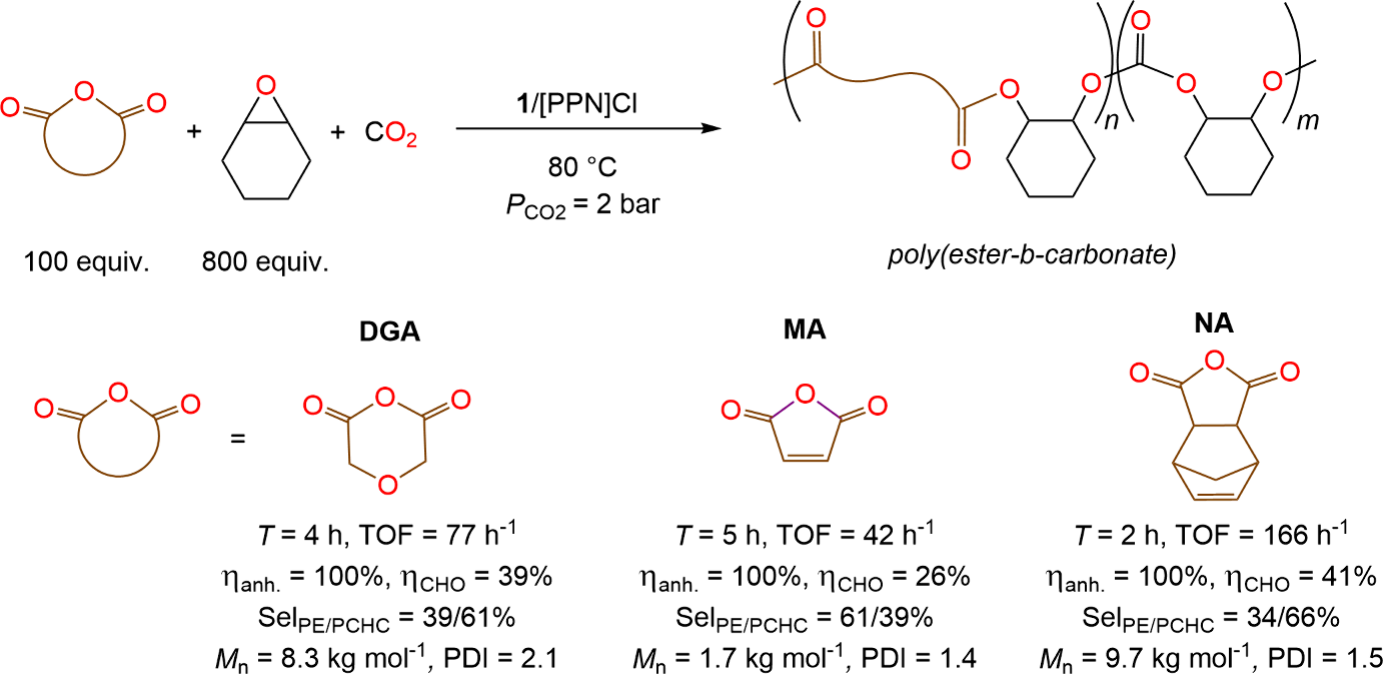
Terpolymerization of CHO with Mono- and Tri-cyclic Anhydrides and CO_2_

Additionally, PO and cyclopentene oxide (CPO) were also tested in catalysis under standard terpolymerization conditions (Table S4, entries 4–5). After 45 min, PO showed 48% conversion to polyester in absence of polycarbonate (Figure S15).^54^ With CPO, 96% of the monomer remained unreacted after 45 min, indicating a very slow rate of polyester formation (Figure S16). Extending the reaction time to 24 h did not lead to the formation of terpolymers.

### Copolymerization of PA and CHO: Activity and Kinetics

Several catalysts capable of efficiently copolymerizing CHO and PA are found in literature.^4,6,14,15^ Among these, heterodinuclear catalysts featuring M­(III)/K (M = Al, Fe)^55–57^ and amino triphenolate based dinuclear-Fe^58^ in the presence of [PPN]Cl have reported excellent activities (TOFs > 1000 h^–1^) and high selectivity toward polyesters. In addition, modified Al/K catalyst also displayed excellent tolerance to copolymerization in the presence of cyclohexene-1,2-diol.^57^ Other active bimetallic systems include dinuclear- Al^59^ and -Cr^60^ catalysts that efficiently copolymerize CHO and PA with TOF up to 1000 h^–1^ at low temperatures (50–60 °C). Monometallic catalysts based on Cr,^30,41,61^ and Zn^62^ reported until now show moderate activity (TOF up to 400 h^–1^) in copolymerization of CHO and PA. So far, only one Ti complex has been reported and displayed moderate activity in copolymerization of PA/CHO (TOF 124 h^–1^).^63^

In light of high activity observed for group 4-NHC catalysts, especially titanium complex **1** in terpolymerization (Table 1, entries 1–3), which is in sharp contrast to the general catalytic trend displayed by these catalysts in epoxide/CO_2_ copolymerization where Zr > Hf ≈ Ti, the polymerization reaction was terminated after 5 min to compare and evaluate the initial rates of ROCOP of CHO and PA for complexes **1**–**3**. Since the resultant terpolymer is a diblock, the high activity of **1** must derive from higher rates of ROCOP of PA and CHO.

Under similar conditions used in terpolymerization study (**1**:[PPN]­Cl:CHO:PA = 1:1:800:100, at 80 °C), complex **1** converts 51% of PA with high activity (TOF = 578 h^–1^) and selectivity (≥99%) toward polyester (Table 3, entry 1). The resulting polymer showed a bimodal distribution with a narrow polydispersity (*Đ* = 1.28) and a low molecular weight (*M*_n_ = 3.9 kg mol^–1^).

**Table 3:** Copolymerization of PA and CHO Catalyzed by Group 4-NHC Complexes^a^

Run	Complex	η_PA_ ^b^ (%)	η_CHO_ ^b^ (%)	TOF (h^–1^)
1	**1**	51	6	578
2	**2**	7	1	96
3	**3**	15	2	192
4^c^	**1**	100	20	1626
5^d^	**1**	100	30	1065
6	**8**	50	6	578
7	**9**	5	1	96

a copolymerization conditions: complex:[PPN]­Cl:CHO:PA = 1:1:800:100, at 80 °C for 5 min.

b Conversion determined by ^1^H NMR spectroscopy of crude mixture.

c **1**:[PPN]­Cl:CHO:PA = 1:1:500:100, 100 °C for 5 min.

d **1**:[PPN]­Cl:CHO:PA = 1:1:2000:400, 100 °C for 15 min.

The bimodal distribution and the low molecular weight is common for this type of copolymerization and mostly induced by the presence of CTAs.^37–42,64–72^ This was further confirmed through end-group analysis of the polyester by GPC-SEC-MS (Figure S17). The mass spectrum revealed three series of ions with different end-groups with a repeating unit of 246.08928 g mol^–1^ consistent with the alternated enchainment of CHO and PA. At low molecular weights approximatively ranging from 1 to 4 kg mol^–1^, two series of ions were determined to correspond to the Cl and O*i*Pr initiated α-ω-chlorohydroxyl and α-ω-isoproxyhydroxyl end-capped PEs, respectively, based on their exact mass in the mass spectrum (Figure S17). The other series of ions observed have molecular weights above ca. 4 kg mol^–1^ and contain α-ω-dihydroxyl end-capped PEs, most likely formed from the chain-transfer reactions by the presence of protic species as residual water and cyclohexane-1,2-diol (Figure S17).^37–42,64–72^ To assess the influence of 1,2-cyclohexenediol as an impurity in the copolymerization of CHO and PA, an excess of 10 equiv was introduced. With this excess, we found that the initiation rate of copolymerization of CHO/PA catalyzed by **1** (**1**:[PPN]­Cl:CHO:PA = 1:1:800:100) at 80 °C is slower with a TOF of 480 h^–1^ (full conv. of PA after 15 min, compared to full conv. at 5 min without diol) and can also occur from the diol, as a majority of the PE contain α-ω-dihydroxyl end-capped PEs determined via GPC-SEC-MS (Figure S18). The GPC-SEC-MS spectrum of diol-initiated PE further revealed a low molecular mass (*M*_n_ = 1.7 kg mol^–1^) and narrow dispersity (*Đ* = 1.05). Moreover, besides most polymeric chains being initiated by 1,2-cyclohexenediol, three minor ion series were detected (Figure S18): cyclic PE and two α-ω-chlorohydroxyl end-capped PEs containing two and three additional cyclohexyloxide units, respectively. This experiment suggests that traces of diol contribute to the bimodal distribution of PE. Furthermore, it demonstrates that chain-end control could be achieved by the presence of diol, predominantly yielding hydroxyl end-capped PE as the major polymer, at the expense of simultaneously decreasing activity and molecular weight and promoting the cyclic PE formation. The decrease in reaction rate is not uncommon for many other catalysts that follow the same trend when excess alcohol is added to the ROCOP of CHO/PA,^38,73–77^ but in sharp contrast with catalytic systems such as heterodinuclear Al/K and YCl_3_(THF/H_2_O)_3_/[PPN]­Cl, for instance, where the rate is accelerated and there is a better end-group control.^57,78^

Thereafter, zirconium and hafnium complexes **2** and **3** were tested in copolymerization of CHO/PA and both catalytic systems displayed a sharp decline in activity compared to titanium complex **1** (Table 3, entries 2–3). The observed trend in catalytic activity Ti ≫ Hf > Zr seems to be influenced by the smaller size of Ti (*r*_Ti_ = 0.74 Å for a 6-coordinate complex), which enhances its Lewis acidity compared to Hf (*r*_Hf_ = 0.85 Å) and Zr (*r*_Zr_ = 0.86 Å). This increased Lewis acidity appears to drive the activation of CHO and PA, thereby facilitating the ring-opening process and thus contributing to the superior catalytic performance of Ti in this context. Despite its high activity in CHO/CO_2_ copolymerization, zirconium complex **2** exhibited the lowest activity in the ROCOP of CHO and PA (TOF = 96 h^–1^). This is noteworthy considering the similarities in both ROCOP pathways.^13,14,17^ Next the performance of complex **1** was evaluated, and at 100 °C, the activity further increases up to 1626 h^–1^ (Table 3, entry 4), marking one of the highest activities achieved for ROCOP of CHO and PA using monometallic binary catalyst systems.^14,15^ Furthermore, at 100 °C, complex 1 also retained high activity with a TOF of 1065 h^–1^ at lower catalyst loading (Table 3, entry 5).

Furthermore, to evaluate the electronic influences of the NHC backbone on the ROCOP of CHO and PA, complexes **8** and **9** bearing benzannulated and unsaturated NHC ligands were tested under standard conditions of terpolymerization (Scheme 3). Complex **8** exhibited activity comparable to that of **1**, whereas **9** displayed a sharp decline in activity. A similar trend observed experimentally in epoxide-CO_2_ copolymerization was attributed to the pronounced donor abilities of saturated and benzannulated NHC ligands in complexes **1** and **8**, respectively, compared to the unsaturated NHC analogue in complex **9**.^33^ Therefore, these effects are likely to contribute to CHO/PA copolymerization catalysis also.

**Scheme 3:**
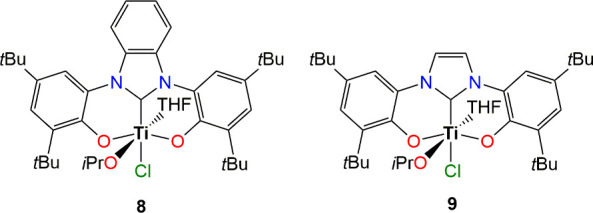
Benzannulated and Unsaturated NHC Complexes of Titanium

To establish the significance of ROCOP of CHO and PA, a reasonable comparison of the catalytic systems based on complex **1** and the known catalysts from the literature was further reviewed. Complex **1** maintained superior activity over other monometallic systems such as (salphen)­CrCl,^41^ (diamino-phenolate)­CrCl,^30^ (amino-triphenolate)­Cr,^61^ (amino-triphenolate)­TiCl^63^ as well as (C_6_F_5_)_2_Zn^62^ reported for CHO/PA copolymerization (TOFs < 380 h^–1^, Table S5, entries 1–5) and functions efficiently under mild reaction conditions compared to organoboron catalysts that demand higher reaction temperatures (120 °C, Table S5, entry 6).^79^ Among the bimetallic systems, the performance of complex **1** is better than (*o*-vanillin)­di-Zn,^23^ but does not outperform other dinuclear systems such as (amino-triphenolate)­di-Fe,^58^ (μ-biphenol)­di-Al^59^ and (salophen)­di-Cr^60^ which require cocatalysts, even though the direct comparison is difficult due to the different reaction conditions (Table S5, entries 8–10). However, the activity of complex **1**, under similar reaction conditions (cat:CHO:PA = 1:2000:400 at 100 °C), is comparable to heterodinuclear systems featuring (*o*-vanillin)­Al/K,^55^ (*o*-vanillin)­Al/Rb^55^ and (*o*-vanillin)­Fe/K^56^ (TOFs = 1072–1152 h^–1^, see Table S5, entries 11–13). Notably, (*o*-vanillin-C_2_Me_2_)­Al/K system display superior activity to complex **1** in the presence of 400 equiv of *trans*-cyclohexane-1,2-diol (TOF = 1890 h^–1^, Table S5, entry 14).^57^

It has been reported that the rate of CO_2_ insertion into M-alkoxide is slower than that for the ring-opening of cyclic anhydrides during terpolymerization.^21^ Thus, it is clear that to form diblock terpolymers, the catalyst significantly lowers the activation barrier for ROCOP of cyclic anhydrides to favor polyesters’ formation over polycarbonates, but reports on direct comparison of these two activation barriers are rare. Previously, in this regard, anionic species [({κ^3^-O,C,O}-NHC)­HfCl_3_], isolated upon addition of [PPN]Cl to hafnium analogue of **4**, have been reported to have a transition-state of activation energy 13.8 ± 0.3 kcal mol^–1^ for CHO/CO_2_ copolymerization.^44^ For temperature-dependent kinetic studies of **1**, conversions of CHO were monitored via *in situ* ^1^H NMR spectroscopy over a temperature range of 70–100 °C (Figure S19). The initial rates obtained from the linear fit were plotted as a function of temperature to establish a transition-state (Δ*H* ^‡^ = 9.4 kcal mol^–1^, Δ*S* ^‡^ = −33 cal mol^–1^ K^–1^) with an activation energy (Δ*E*_a_) of 10.0 kcal mol^–1^ (Figures 3 and S20). The Gibbs free energy at 80 °C (Δ*G* ^*‡*^) was calculated to be 21.1 kcal mol^–1^. The transition-state of **3** (Δ*H* ^*‡*^ = 10.7 kcal mol^–1^, Δ*S* ^*‡*^ = −29 cal mol^–1^), was found to have an activation energy of 11.5 kcal mol^–1^ and an overall Δ*G* ^*‡*^ of 21.2 kcal mol^–1^ (Figures S21 and S22). The determined energetics of **3** agree well with the reported data in CHO/CO_2_ copolymerization as well as account for the lower activity compared to **1** in CHO/PA copolymerization. The results are also consistent with the kinetic data for monometallic Cr salen system reported for CHO/PA copolymerization (Δ*G* ^*‡*^ ≈ 24 kcal mol^–1^ at 80 °C).^27,29,60^

**Figure 3:**
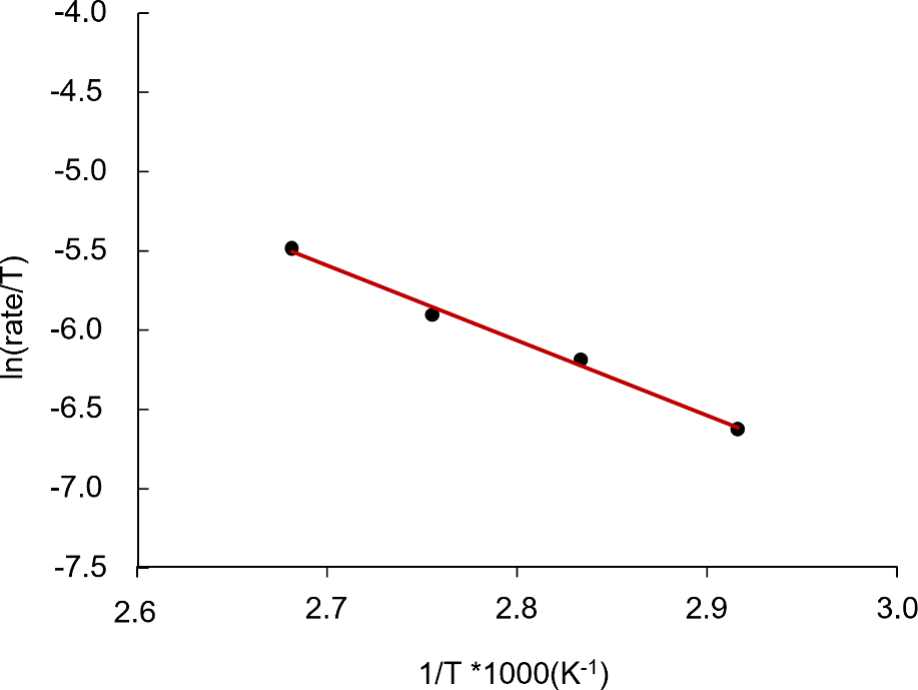
Eyring’s plot for ROCOP of PA and CHO catalyzed by **1**/[PPN]­Cl.

## Conclusions

Highly efficient bis-phenolate group 4 NHC catalysts showing high activity (TOFs up to 460 h^–1^) in the presence of [PPN]Cl for terpolymerization of CHO, PA and CO_2_ leading to poly­(ester-*b*-carbonate)­s were reported for the first time. The most active system consisting of titanium complex **1**/[PPN]Cl also successfully terpolymerized mono-, bi-, and tricyclic anhydrides to yield polyester modulated diblock terpolymers. The catalytic efficiency of titanium in the terpolymerization of PA and CHO with CO_2_ is directly linked to its unprecedented activity observed experimentally in ROCOP of CHO and PA and is consistent with the low activation barrier determined via temperature-dependent kinetic studies. This study opens up a new impetus for group 4-based catalysts illustrating their potential in terpolymerization as well as copolymerization catalysis.

## Experimental Section

### General Considerations

All experiments were carried out under an atmosphere of argon employing standard Schlenk and glovebox techniques, unless otherwise stated. **Caution!** *Extreme care should be taken both in the handling of the cryogen liquid nitrogen and its use in the Schlenk line trap to avoid the condensation of oxygen from air.* Bis­(triphenylphosphine)­iminium salt [PPN]Cl was recrystallized from dichloromethane/hexane (1/3), dried several hours at 80 °C under vacuum.^80^ [*n*Bu_4_N]­Cl/Br, DMAP and PPh_3_ were dried under at 80 °C under vacuum before transferring to glovebox. Cyclohexene oxide was purchased from Sigma-Aldrich, distilled over CaH_2_ under vacuum following three freeze–pump–thaw cycles, and stored at −30 °C in a glovebox. All cyclic anhydrides were purchased from Sigma-Aldrich and dried under prolonged vacuum before use. CO_2_ (99.999%) from Praxair was purified through CuO on alumina and molecular sieves (3 Å).

### Synthesis of ({κ^3^-*O*,*C*,*O*}-NHC)­Ti­(OAc^F^)_2_ (**7**)

To a solution of complex **4** (30 mg, 0.05 mmol) in dichloromethane was added 2 equiv of NaOAc^F^ (13.7 mg, 0.1 mmol) suspended in dichloromethane (5 mL) and stirred at room temperature. After stirring overnight, the reaction mixture was centrifuged, filtered through a filter pipet, and dried under vacuum to yield a greenish powder (yield = 90%). Pale green crystals of **7** were obtained by layering a solution of **7** in chloroform with hexane (1/3) at −30 °C. ^1^H NMR (500.13 MHz, benzene-*d*_6_): δ 7.42 (d, *J* = 2.2 Hz, 2H, Ar-*H*), 6.69 (d, *J* = 2.2 Hz, 2H, Ar-*H*), 3.07 (s, 4H, NC*H*_2_), 1.60 (s, 18H, *t*Bu), 1.37 (s, 18H, *t*Bu) ppm. ^13^C­{^1^H} NMR (125.75 MHz, benzene-*d*_*6*_): δ 196.3 (N*C*N), 173.1 (CH_3_ *C*(O)­O), 149.9 (*C*_q_, Ar), 143.5 (*C*_q_, Ar), 137.6 (*C*_q_, Ar), 132.7 (*C*_q_, Ar), 118.9 (*C*H, Ar), 111.8 (*C*H, Ar), 46.7 (N*C*H_2_), 35.5 (*C*_q_, *t*Bu), 34.6 (*C*_q_, *t*Bu), 31.5 (*C*H_3_, *t*Bu), 29.5 (*C*H_3_, *t*Bu) ppm. ^19^F NMR (470.70 MHz, benzene-*d*_*6*_): δ −77.6 ppm. DRIFT (KBr, ν/cm^–1^): 2960s, 2907m, 2872m, 1727s, 1678m, 1541m, 1479vs, 1452vs, 1393w, 1325w, 1202m, 1174w, 941w, 868w, 796w, 736w, 758w, 736w, 719w, 673w. Anal. Calcd for C_35_H_44_F_6_N_2_O_6_Ti.1.5CH_2_Cl_2_: C, 49.93; H, 5.40; N, 3.19%. Found: C, 50.58; H 5.31; N 3.19%.

### Representative Procedures for the Terpolymerization and Copolymerization

In a glovebox, a high-pressure reactor was loaded with complex **1** (8 μmol, 1 equiv) and a solution of [PPN]Cl (8 μmol, 1 equiv) in CH_2_Cl_2_ (1 mL). The mixture was allowed to react for 20 min and then dried under a vacuum. To this solid mixture, phthalic anhydride (0.8 mmol, 100 equiv) and precooled CHO at −30 °C (0.65 mL, 6.4 mmol, 800 equiv) were added. For the terpolymerization, the reactor was then pressurized to 2 bar of CO_2_. After heating up to 80 °C for 45 min in an oil bath (terpolymerization) or for 5 min in a heating block (copolymerization), the reactor was cooled to room temperature. An aliquot of the crude mixture was collected and analyzed by ^1^H NMR spectroscopy in chloroform-*d*. The reaction was then quenched using 1 mL of 5% acidified methanol, and the precipitated polymer was dried at 80 °C for 4 h under vacuum.

### Representative Procedures for the Kinetic Studies for ROCOP of PA and CHO

In a glovebox, a vial was loaded with complex **1** (8 μmol, 1 equiv) and a solution of [PPN]Cl (8 μmol, 1 equiv) in CH_2_Cl_2_ (1 mL) and allowed to react for 15 min. The resulting mixture was evaporated to dryness, and 100 equiv of PA (0.8 mmol) and 800 equiv of CHO (0.65 mL, 6.4 mmol) were added to the vial. Then, the reaction mixture was heated to the required temperature (heating block). An aliquot of the crude mixture was collected at regular time intervals, to monitor the polyester formation by ^1^H NMR spectroscopy. The NMR conversions were plotted against time, and the slope of the linear fit was used to derive the reaction rate.

## Supplementary Material



## Data Availability

The data underlying this study are available in the published article and its Supporting Information.

## Notes

General methods, NMR spectra, X-ray crystallographic data, GPC-SEC, DSC, TGA and MS for polymers, and kinetic plots (PDF)
