# Supplementary material for: N‑Heterocyclic Carbene-Based Group 4 Catalysts for the Terpolymerization of Cyclohexene Oxide and Cyclic Anhydrides with CO2
Source: ACS Org Inorg Au. 2025 May 13;5(3):171–80. doi: 10.1021/acsorginorgau.5c00002 (PMC12142441; doi:10.1021/acsorginorgau.5c00002)
Supplement: Supplementary file 1 [file gg5c00002_si_001.pdf]

Supporting Information

N-Heterocyclic Carbene-Based Group 4 Catalysts for the Terpolymerization of Cyclohexene Oxide and Cyclic Anhydrides with CO_2

Lakshmi Suresh,[a] Kathrin Zwettler,[a] Karl W. Törnroos,[a] William Le,[b] Benoît Marcolini,[c] Gilles Frache,[b] Erwan Le Roux[a]*

[a] Department of Chemistry, University of Bergen, Allégaten 41, N-5007, Bergen, Norway.

[b] Luxembourg Institute of Science and Technology (LIST), 41, rue du Brill, L-4422, Belvaux, Luxembourg.

[C] Luxembourg Institute of Science and Technology (LIST), 41, rue du Brill, L-4422, Belvaux, Luxembourg.

E-Mail: Erwan.LeRoux@uib.no

TABLE OF CONTENTS

General Methods S3

Figure S1. ^{1}H NMR Spectrum of Complex **7** S4

Figure S2. ^{13}C{^{1}H} NMR Spectrum of Complex **7** S5

Figure S3. ^{19}F NMR Spectrum of Complex **7** S5

Figure S4. ^{1}H-^{1}H COSY and ^{1}H-^{13}C HSQC NMR Spectra **7** S6

Table S1. Crystal Structure and Refinement Data for**7** S7

Figure S5. ^{1}H NMR Spectrum of Crude Terpolymer of PA, CHO and CO_2 S8

Figure S6. ^{1}H NMR Spectrum of Isolated Terpolymer of PA, CHO and CO_2 S8

Figure S7. ^{13}C{H} NMR Spectrum of Isolated Terpolymer of PA, CHO and CO_2 S9

Figure S8. GPC-SEC Profile of Terpolymer of PA, CHO and CO_2 S9

Table S2. Effect of Cocatalysts on Terpolymerization of CHO, PA and CO_2 S10

Table S3. Summary of Thermal Properties of Poly(ester-*b*-carbonate)s S10

Figure S9. DSC Thermograms of Poly(ester-*b*-carbonate)s S11

Figure S10. TGA Thermograms of Poly(ester-*b*-carbonate)s S11

Table S4. Terpolymerization Using Various Cyclic Anhydrides and Epoxides S12

Figure S11. ^{1}H NMR Spectrum of the Terpolymerization of DGA, CHO and CO_2 S12

Figure S12. ^{1}H NMR Spectrum of the Terpolymerization of MA, CHO and CO_2 S13

Figure S13. ^{1}H NMR Spectrum of the Terpolymerization of NA, CHO and CO_2 S13

Figure S14. GPC-SEC Profile of Terpolymer of DGA, MA and NA, CHO and CO_2 S14

Figure S15. ^{1}H NMR Spectrum of the Terpolymerization of PA, PO and CO_2 S14

Figure S16.^{1}H NMR Spectrum of the Terpolymerization of PA, CPO and CO_2 S15

Figure S17. Average Mass Spectra from GPC-SEC-MS of Polyester S15

Figure S18. Average Mass Spectra from GPC-SEC-MS of Polyester in Presence of Diol S16

Table S5. Selected Examples of Catalysts for CHO/PA Copolymerization S17

Scheme S1. Structure of the Complexes Copolymerizing CHO/PA S18

Figure S19. Overlay of CHO Conv. vs. Time Plots for ROCOP of CHO and PA S19

Figure S20. Arrhenius Plot for ROCOP of PA and CHO Catalyzed by **1** S19

Figure S21. Arrhenius Plot for ROCOP of PA and CHO Catalyzed by **3** S20
Figure S22. Eyring's Plot for ROCOP of PA and CHO Catalyzed by **3** S20
References S21

General procedures. All experiments were performed under inert atmosphere using standard Schlenk, high-vacuum and glovebox techniques (MB Braun MB200B-G; < 1 ppm O_2, < 1 ppm H_2O). Solvents like diethyl ether, dichloromethane, were dried over CaH_2, toluene pentane and hexane were purified over a sodium mirror and THF was distilled from sodium benzophenone ketyl (pre-purified over Grubbs columns: MB Braun Solvent Purification System 800). All solvents were vacuum distilled, degassed and filtered prior to use. Benzene-d_6 and chloroform-*d* were obtained from Aldrich, dried over sodium or CaH_2, vacuum distilled, degassed, and filtered prior to use. All other chemicals were purchased from Sigma-Aldrich and used as received. The NMR spectra of air and moisture sensitive compounds were recorded using J. Young valve NMR tubes at 25 ˚C on Bruker BioSpin AVANCE AV500 (5 mm BBO, ^{1}H: 500.13 MHz; ^{13}C: 125.75 MHz), Bruker AVANCE NEO 600 spectrometer equipped with a QCI-P CryoProbe (^{1}H: 600.13 MHz; ^{13}C: 150.90 MHz) and Bruker BioSpin 500 widebore ascend spectrometers (5 mm BBO with z-gradient BBI, ^{19}F NMR: 470.70 MHz). Chemical shifts are referenced to the residual proton solvent signals (^{1}H: chloroform-*d*, δ 7.26; benzene-d_6: 7.16 ppm) and solvent ^{13}C signals (^{13}C: chloroform-*d*, δ 77.16; benzene-d_6: 128.06 ppm) and are reported in *parts per million* (ppm) relative to tetramethylsilane.[1] ^{19}F signal was referenced to lock frequency and are given relative to the CCl_3F in benzene-d_6. IR spectra were recorded on a Nicolet FT-IR Protégé 460 spectrometer with a DRIFT collector. The spectra averaged over 64 scans; the resolution was ±4 cm^{-1}. Elemental analyses of C, H and N were performed on an Elementar Vario EL III instrument.

The molecular weights and PDIs were determined by GPC-SEC from Viscotek. Polystyrene PS-99K (M_w = 98 kg mol^{-1}, IV = 0.477) calibration standard for molecular weight calibration were obtained from Viscotek™ (PolyCAL TDS-PS standards). Chromatographic separation of polycarbonates samples was performed at a column temperature of 40 °C with a flowrate of 1 mL min^{-1}. SEC was performed with a pump supplied by Viscotek (GPCmax), employing two ViscoGelTM columns (GMHHR-H columns (300 (L) x 7.8 mm (I.D.), 10 µm particle size, 100 Å pore size). Signals were calibrated against polystyrene standards (M_w/M_n < 1.15, from 1-400 kg mol^{-1}). The NMR conversion and microstructure of the polycarbonates were determined by means of ^{1}H and ^{13}C NMR spectroscopy using chloroform-*d* as solvent.

GPC-SEC-MS was performed using a Thermo Ultimate 3000 HPLC system coupled with a Thermo linear trap quadrupole (LTQ)/Orbitrap Elite high-resolution mass spectrometer equipped with a heated electrospray ionisation interface (Thermo-Fisher Scientific, USA). Chromatographic separation was performed in THF at a column temperature of 30 °C with a flowrate of 1 mL min^{-1} employing two Agilent columns in series (MesoPore column (300 (L) x 7.5 mm (I.D.), 3 µm particle size, 100 Å pore size), and OligoPore column (300 (L) x 7.5 mm (I.D.), 6 µm particle size, 100 Å pore size). Methanol was added prior to the ionization source to promote the formation of ions. Mass spectra were recorded in the 1000-4000 m/z range in positive ion mode.

Differential scanning calorimetry (DSC) analysis was performed on a Netzsch DSC 204 F1 Phoenix instrument using cyclic heating and cooling rates of 10 °C min^{-1} and heated from - 40 to 230 °C. The values of glass transition temperature T_g were recorded during the second thermal cycle. TGA experiments were performed on a Mettler TGA2 STARe System (Mettler Toledo), from 25 °C to 800 °C, at a speed of 10 °C min^{-1} and a nitrogen flow of 40 mL min^{-1}.

X-ray crystallography and crystal structure determination. Suitable crystals for diffraction experiments were selected in a glovebox and mounted in a minimum of Parabar 10312 oil (Hampton Research) in a nylon loop and then mounted under a nitrogen cold stream from an

Oxford Cryosystems 700 series open-flow cryostat. Data collection was done on a Bruker AXS TXS rotating anode system with an APEXII Pt[135] CCD detector using graphite-monochromated Mo Kα radiation (λ = 0.71073 Å). Data collection and data processing were done using APEX2,[2] SAINT,[3] and SADABS[4] version 2012/1, whereas structure solution and final model refinement were done using SHELXT [5] version 2014/4 and SHELXL[6] version 2014/7. CCDC reference code 2393896 contains the supplementary crystallographic data for **7**. These data can be obtained free of charge from The Cambridge Crystallographic Data Centre via www.ccdc.cam.ac.uk/data_request/cif.

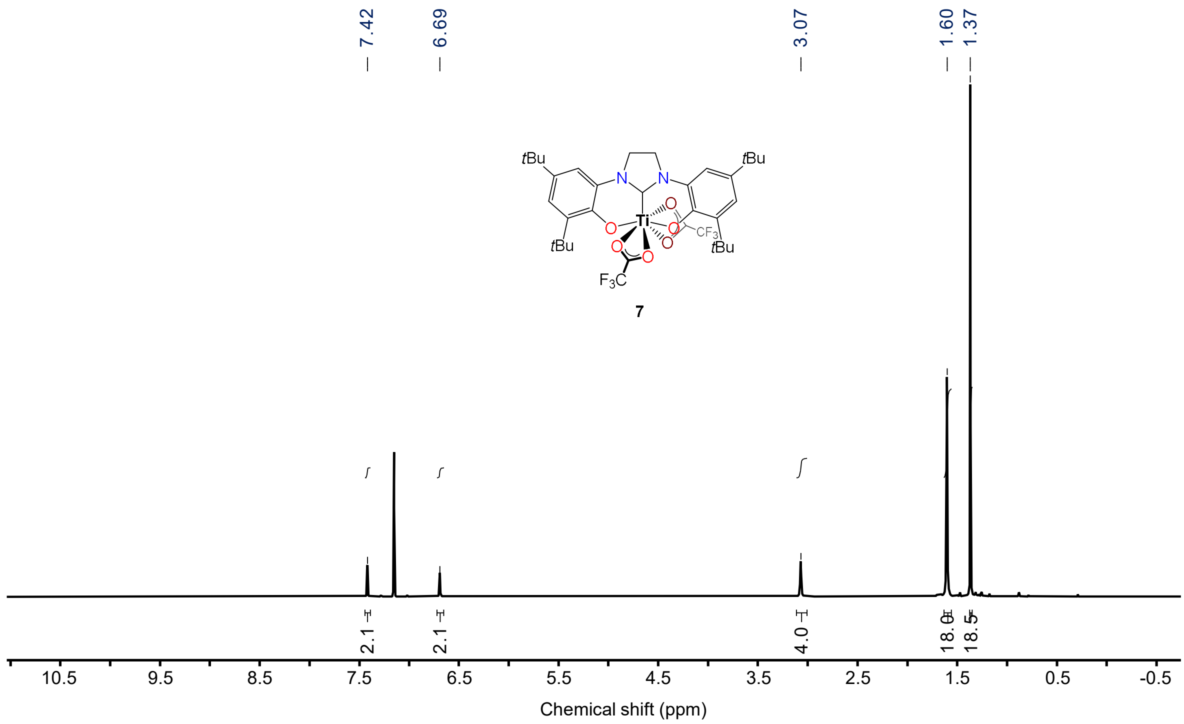

Figure S1. ^{1}H NMR spectrum (298 K, 500.13 MHz) of complex **7** in benzene-d_6.

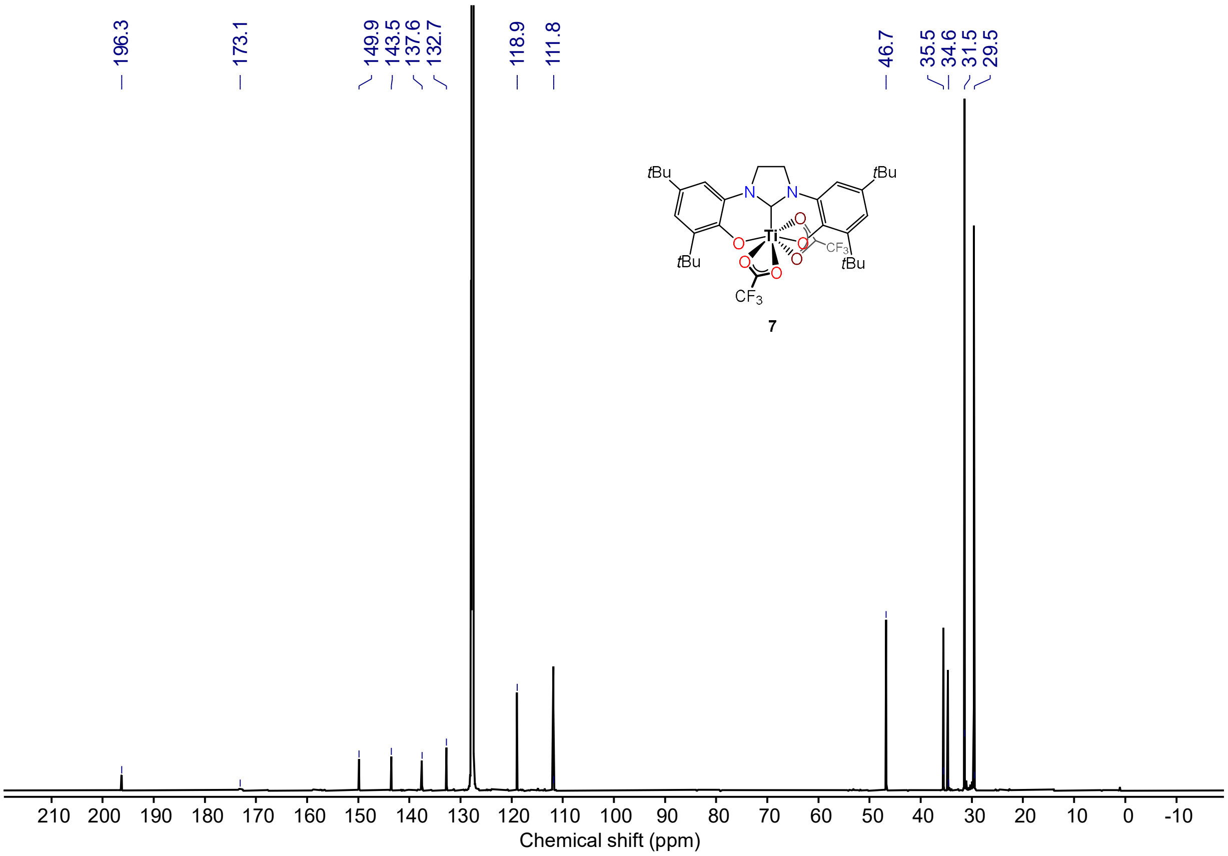

Figure S2. $^{13}C\{^1H\}$ NMR spectrum (298 K, 125.75 MHz) of complex **7** in benzene-d_6.

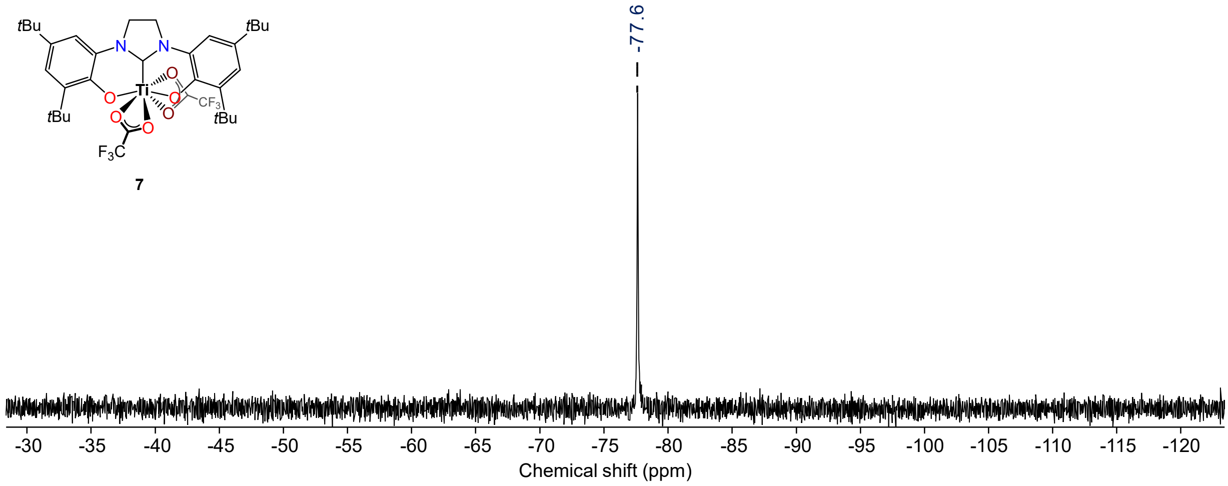

Figure S3. ^{19}F NMR spectrum (298 K, 470.70 MHz) of complex **7** in benzene-d_6.

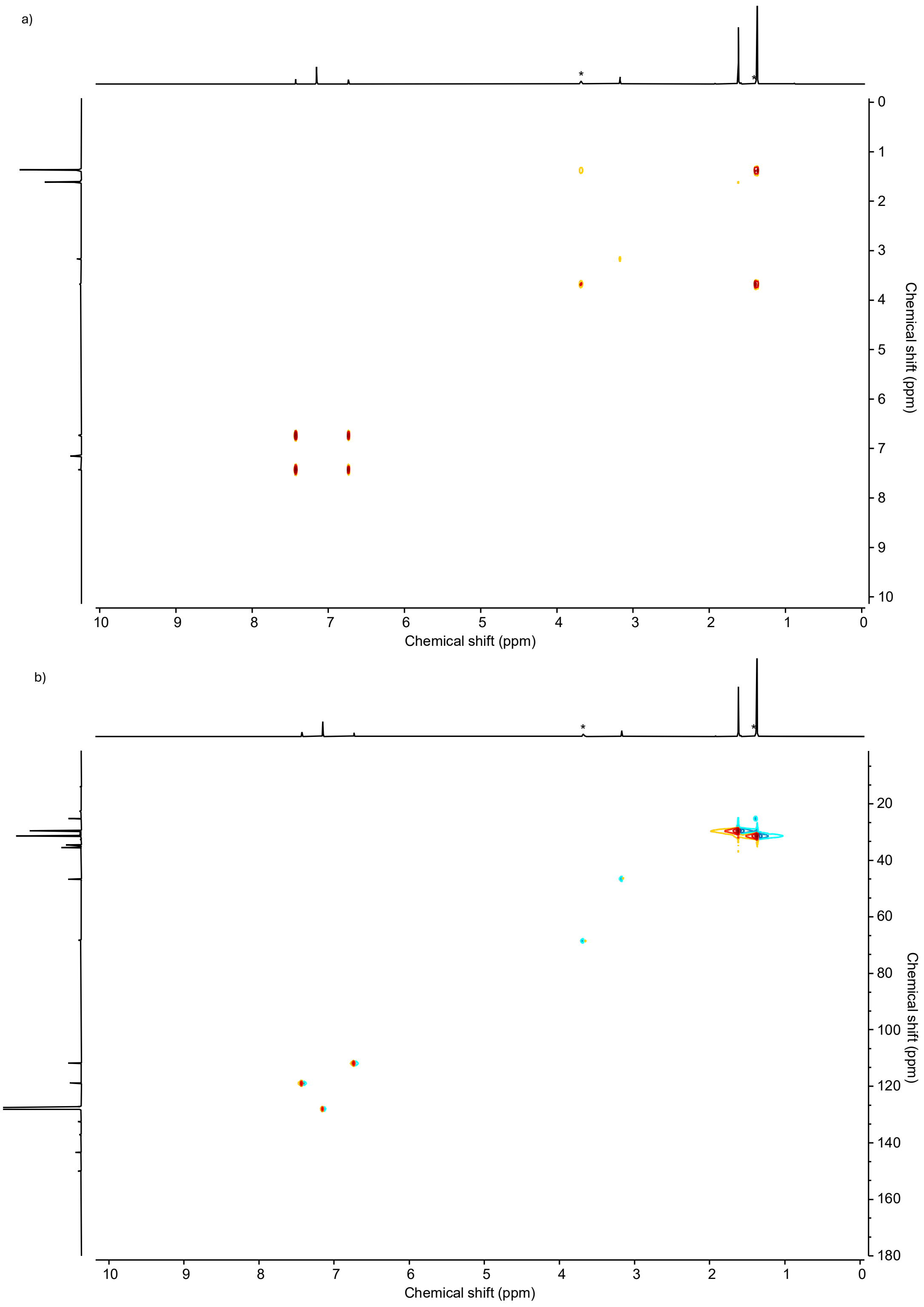

Figure S4. a) ^{1}H-^{1}H COSY NMR spectrum, and b) ^{1}H-^{13}C HSQC NMR spectrum (298 K, 600.13 MHz) of complex **7** in benzene-d_6, (* = trace of residual THF).

Table S1. Crystal Data and Structure Refinement for Complex 7.

**Empirical formula**	$\mathbf{C_{35}H_{44}F_6N_2O_6Ti}$
Formula weight	750.62
Temperature	123(2) K
Wavelength	0.71073 Å
Crystal system	Monoclinic
Space group	$P2_1/c$
Unit cell dimensions	a = 14.6237(9) Å
	b = 17.0517(10) Å
	c = 17.7764(10) Å
	α= 90°
	β= 105.4470(10)°
	γ = 90°
Volume	4272.6(4) $Å^3$
Z	4
Density (calculated)	1.167 mg/m^3
Absorption coefficient	0.265 mm^{-1}
F(000)	1568
Crystal size	0.25 x 0.30 x 0.40 mm^3
Crystal colour/Habit	Purple/Block
Theta range for data collection	2.377 to 30.040°.
Index ranges	-18<=h<=20, -23<=k<=23, -25<=l<=25
Reflections collected	45634
Independent reflections	12488 [R(int) = 0.0404]
Completeness to theta = 25.242°	99.9 %
Absorption correction	Semi-empirical from equivalents
Refinement method	Full-matrix least-squares on F^2
Data / restraints / parameters	12488 / 657 / 519
Goodness-of-fit on F^2	1.050
Final R indices [I>2sigma(I)]	R1 = 0.0589, wR2 = 0.1686
R indices (all data)	R1 = 0.0812, wR2 = 0.1918
Extinction coefficient	n/a
Largest diff. peak and hole	0.641 and -0.415 $e.Å^{-3}$

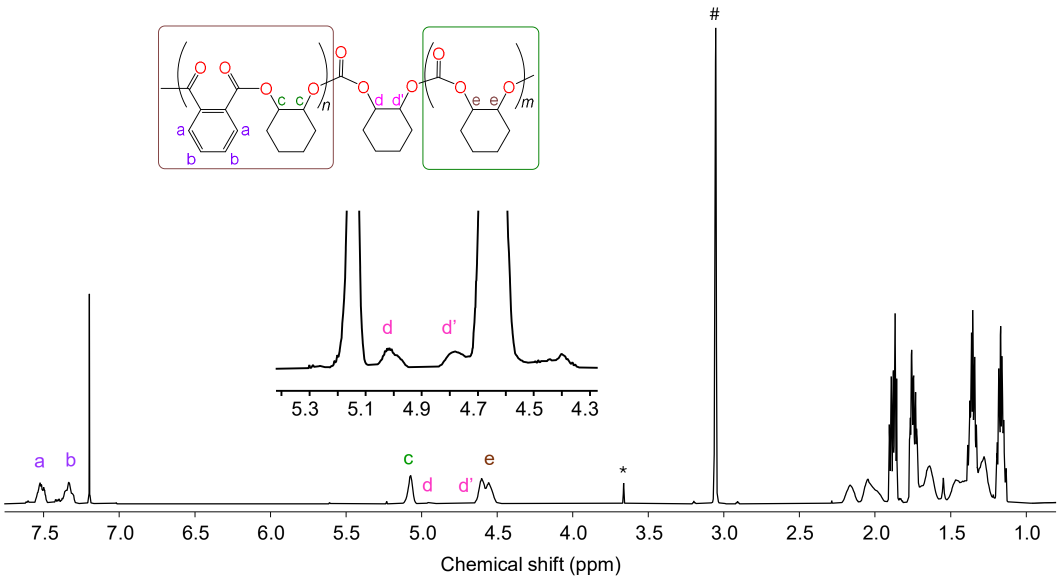

Figure S5. ^{1}H NMR spectrum (298 K, 600.13 MHz) of crude terpolymer of PA, CHO and CO_2 in chloroform-*d* (Entry 1, Table 1, * = impurity, # = CHO).

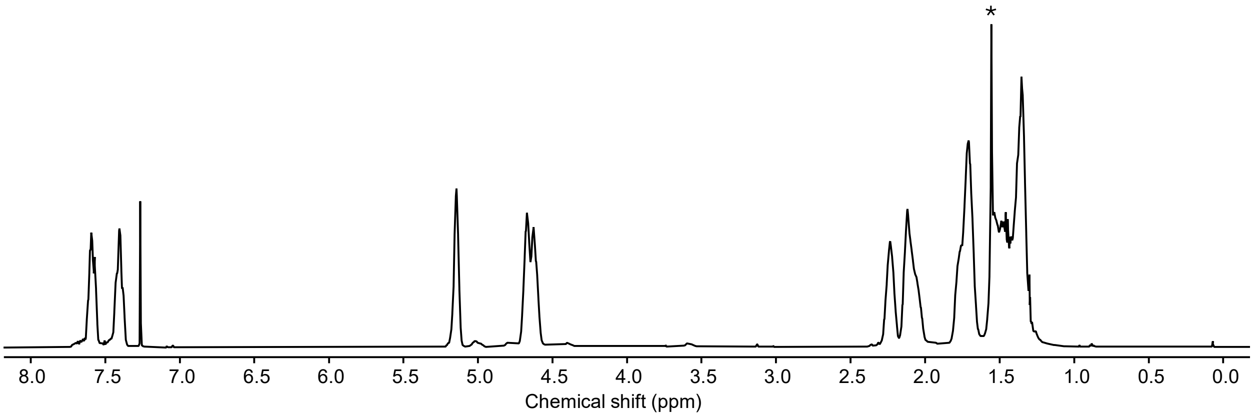

Figure S6. ^{1}H NMR spectrum (298 K, 600.13 MHz) of isolated terpolymer of PA, CHO and CO_2 in chloroform-*d* (Entry 1, Table 1, * = residual water).

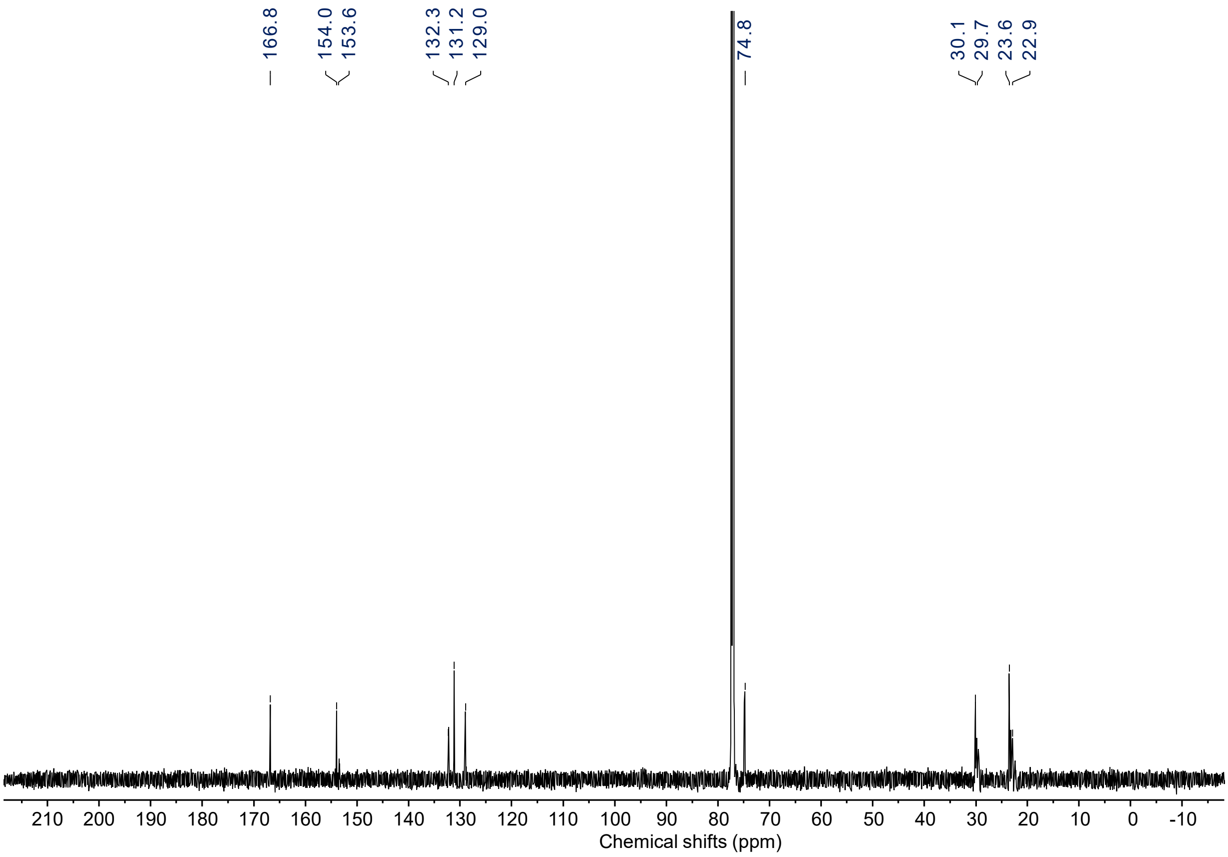

Figure S7. $^{13}C\{H\}$ NMR spectrum (298 K, 125.75 MHz) of isolated terpolymer of PA, CHO and CO_2 in chloroform-*d* (Table 1, Entry 1).

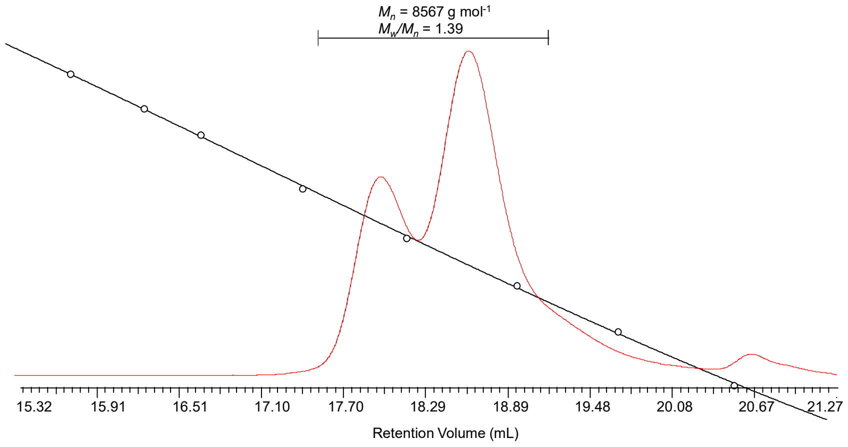

Figure S8. GPC-SEC profile of terpolymer of PA, CHO and CO_2 (Entry 1, Table 1).

Table S2. Effect of Co-catalysts on Terpolymerization of CHO, PA and CO_2 in Presence of 1.

Run[a]	Catalyst	η_{PA}[b] (%)	η_{CHO}[b] (%)	$Sel_{PE/PCHC}$[b] (%)	TOF (h^{-1})
1	**1**/[PPN]Cl	100	40	36/64	429
2	**1**/-	43	6	15/-[c]	69
3	-/[PPN]Cl	70	10	100/-	106
4	**1**/[*n*Bu_4N]Br	100	14	≥99/-	147
5	**1**/[*n*Bu_4N]Cl	87	12	≥99/-	131
6	**1**/DMAP	100	15	≥99/-	158
7	**1**/PPh_3	24	2	≥99/-	24

[a] Terpolymerization conditions: **1**:cocat:CHO:PA = 1:1:800:100. P_{CO2} = 2 bar at 80 °C for 45 min. [b] Conversion and selectivity determined by ^{1}H NMR spectroscopy of crude mixture. [c] 85% polyether content present in the reaction mixture.

Table S3. Summary of Thermal Properties of Poly(ester-*b*-carbonate)s with Various Content of Polyester (PE) and Polycarbonate (PCHC) Catalyzed by 1.[a]

Run[a]	Ratio CHO:PA	η_{PA}/η_{CHO} (%)[b]	PE (%)[b]	PCHC (%)[b]	M_n[c] (kg mol^{-1})	$Đ$[c]	T_g[d] (°C)	T_d (°C)
1	1000:200	100/24	99	1	12.0	1.1	120	303
2	1000:50	100/21	74	26	6.1	1.2	108	205
3	600:100	100/36	57	43	12.7	1.2	107	239
4	800:100	100/40	36	64	12.5	1.2	121	291
5	800:-	-/20	-	>99	8.2	1.1	73	192
6	1250:-	-/100	-	>99	21.0	1.3	118[e]	-

[a] Terpolymerization conditions: P_{CO2} = 2 bar at 80 °C for 45 min (see Table 2 for further details). [b] Conversion and selectivity determined by ^{1}H NMR spectroscopy of crude mixture. [c] Determined by GPC-SEC in THF at 30 °C against polystyrene standard. [d] 2nd heating. [e] Measured T_g value for the PCHC reported in Ref. [7], copolymerization conditions: P_{CO2} = 1 bar at 60 °C for 18 h.

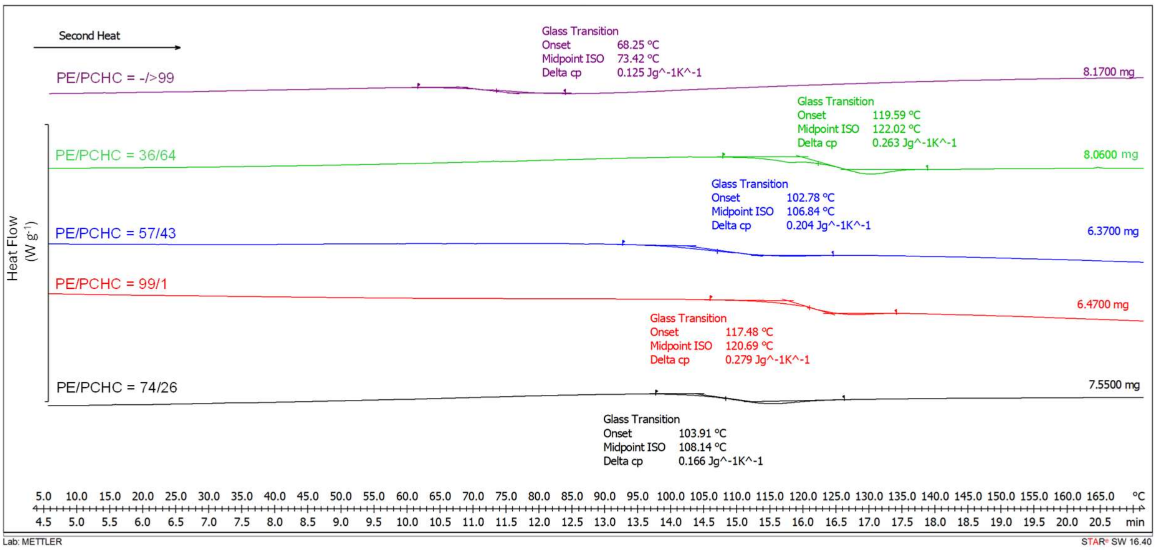

Figure S9. Differential Scanning Calorimetry (DSC) Thermograms of Poly(ester-*b*-carbonate)s with Various Content of Polyester (PE) and Polycarbonate (PCHC).

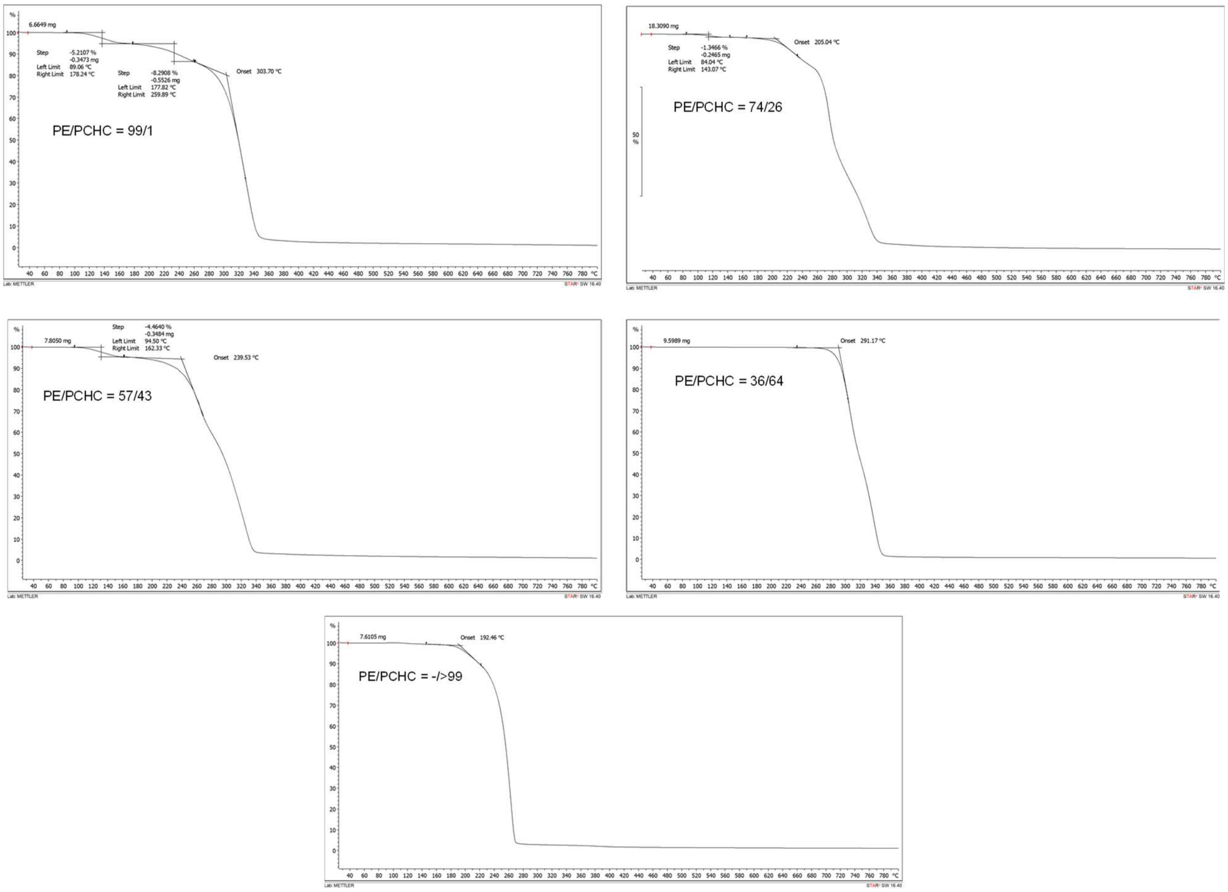

Figure S10. Thermogravimetry Analysis (TGA) Thermograms of Poly(ester-*b*-carbonate)s with Various Content of Polyester (PE) and Polycarbonate (PCHC).

Table S4. Terpolymerization Catalyzed by 1/[PPN]Cl using Various Cyclic Anhydrides and Epoxides with CO_2.[a]

Run	Anhy.	Epox.	Time (h)	η_{anhy}[b] (%)	η_{epox}[b] (%)	$Sel_{PE/PCHC}$[b] (%)	M_n[c] (kg mol^{-1})	$Đ$[c]
1	DGA	CHO	4	100	39	39/61	5.7	2.1
2	MA	CHO	5	100	26	61/39	1.7	1.4
3	NA	CHO	2	100	41	34/66	9.7	1.5
4	PA	PO	0.75	100	48	≥99/-	8.3	1.4
5	PA	CPO	0.75	25	4	≥99/-	-[d]	-[d]

[a] Terpolymerization conditions: **1**:[PPN]Cl:Anhy:Epox = 1:1:100:800, P_{CO2} = 2 bar at 80 °C. [b] Conversion and selectivity determined by ^{1}H NMR spectroscopy of crude mixture. [c] Determined by GPC-SEC in THF at 30 °C against polystyrene standard. [d] Not determined.

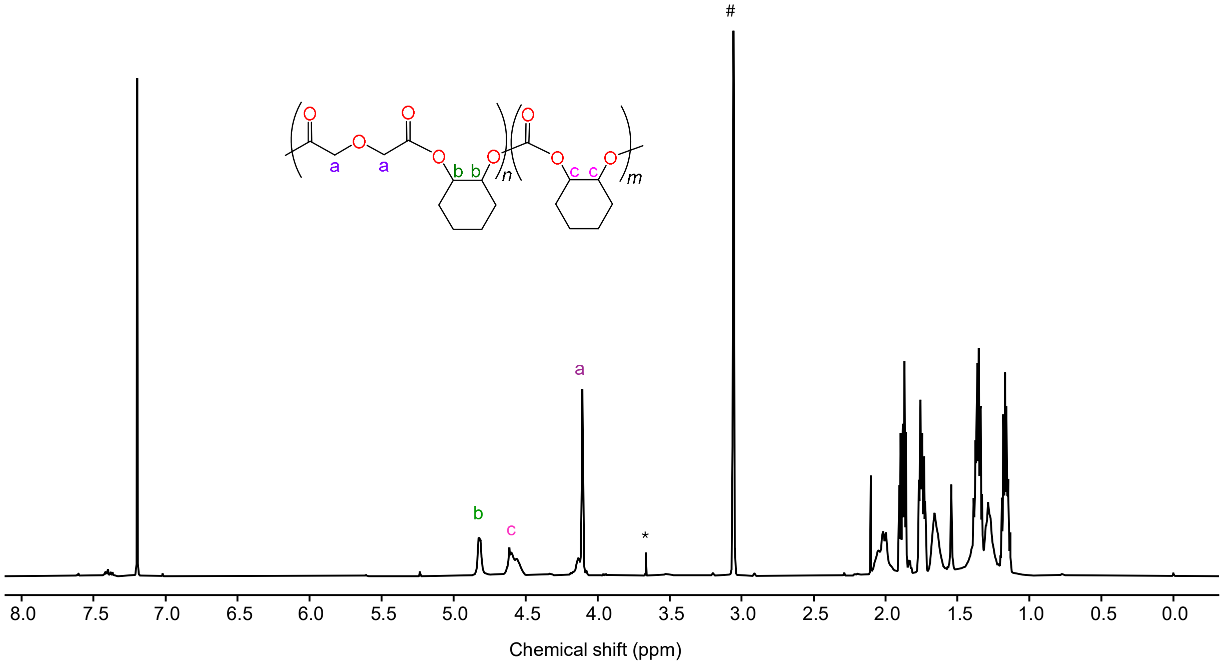

Figure S11. ^{1}H NMR spectrum (298 K, 600.13 MHz) of the terpolymerization of DGA, CHO and CO_2 using **1** in chloroform-*d* (Entry 1, Table S3, * = impurity, # = CHO).[8]

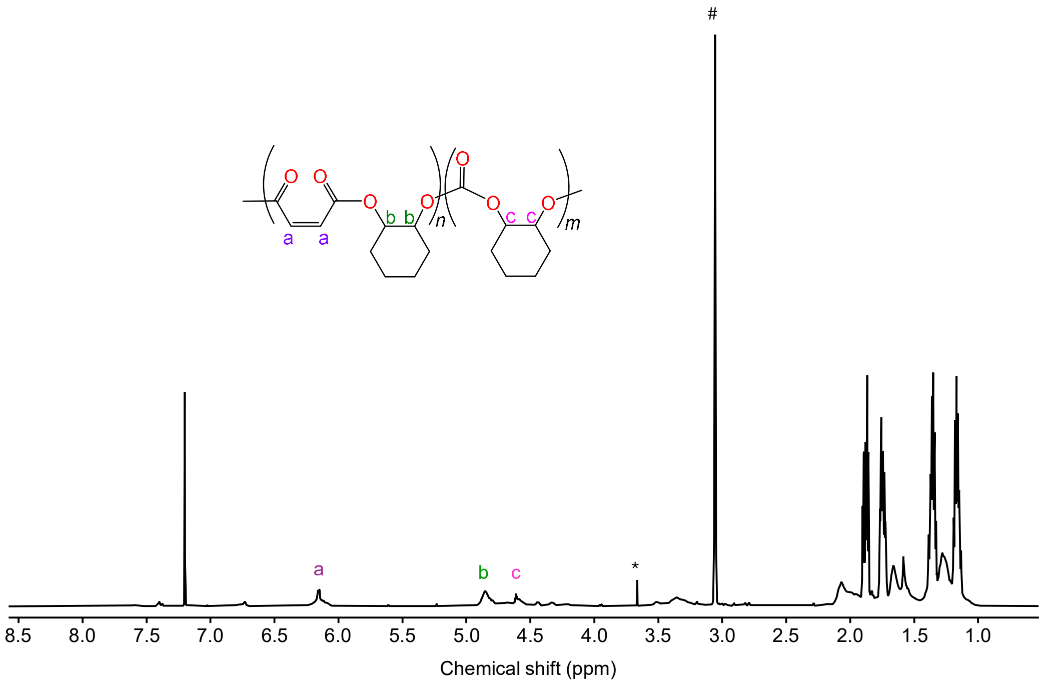

Figure S12. ^{1}H NMR spectrum (298 K, 600.13 MHz) of the terpolymerization of MA, CHO and CO_2 using in chloroform-*d* **1** (Entry 2, Table S3, * = impurity, # = CHO).[9]

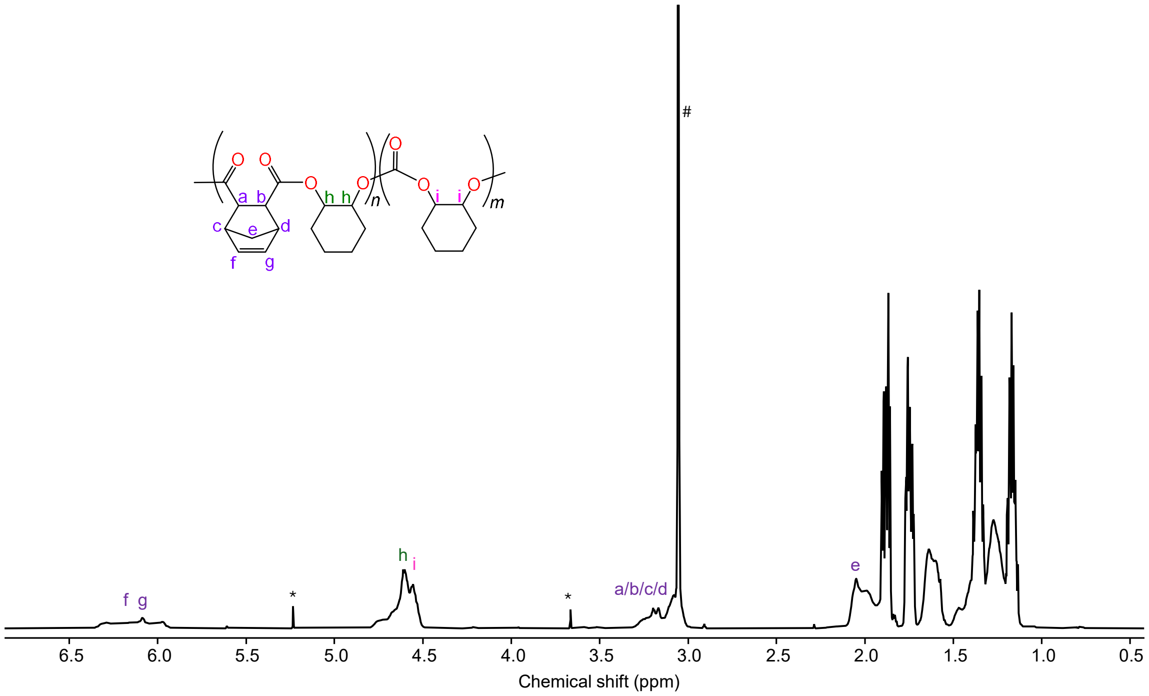

Figure S13. ^{1}H NMR spectrum of the terpolymerization of NA, CHO and CO_2 using **1** in chloroform-*d* (Entry 3, Table S3, * = impurity, # = CHO).[10]

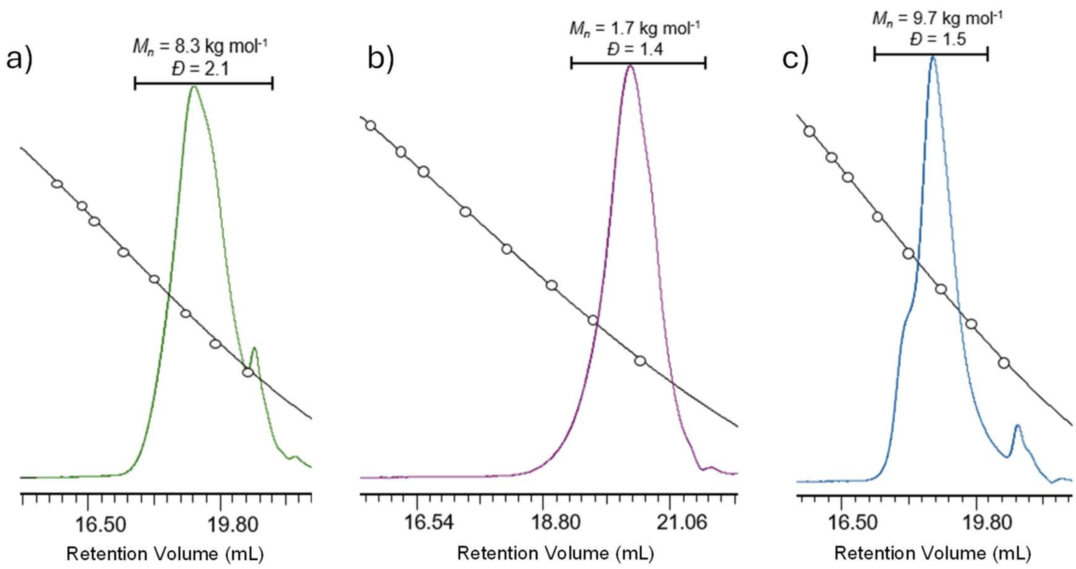

Figure S14. GPC-SEC profile of terpolymer of a) diglyconic anhydride (DGA), b) maleic anhydride (MA) and c) norbornene anhydride (NA) in presence of CHO and CO_2 (Entries 1-3, Table S3).

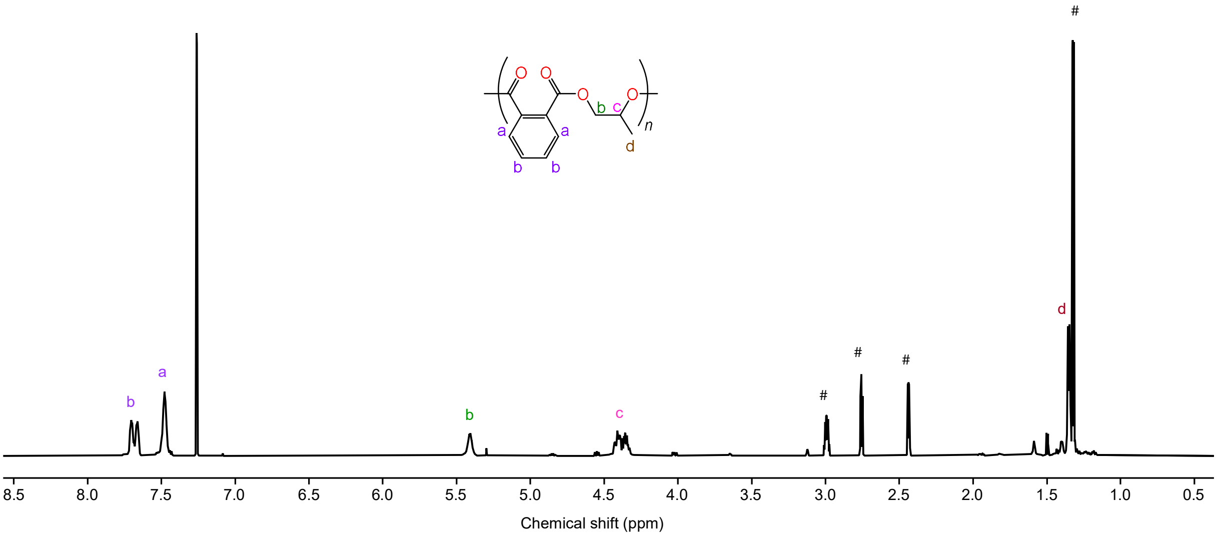

Figure S15. ^{1}H NMR spectrum (298 K, 600.13 MHz) of the terpolymerization of PA, PO and CO_2 using **1** in chloroform-*d* (Entry 4, Table S3, # = PO).[11]

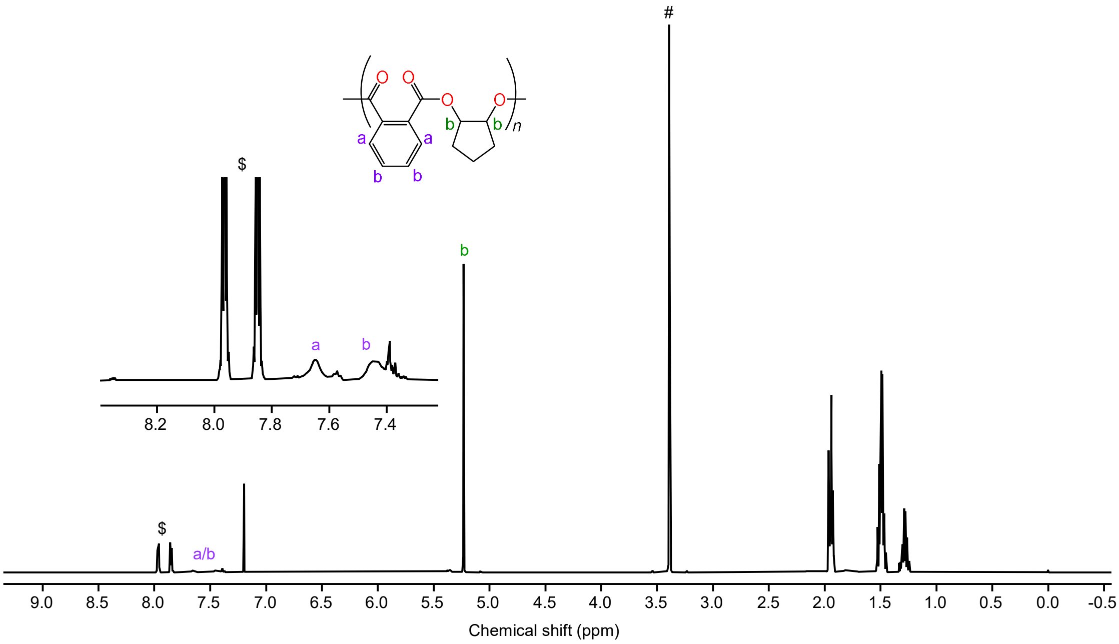

Figure S16. ^{1}H NMR spectrum (298 K, 600.13 MHz) of the terpolymerization of PA, CPO and CO_2 using **1** in chloroform-*d* (Entry 5, Table S3, # = CPO, $ = PA).[12]

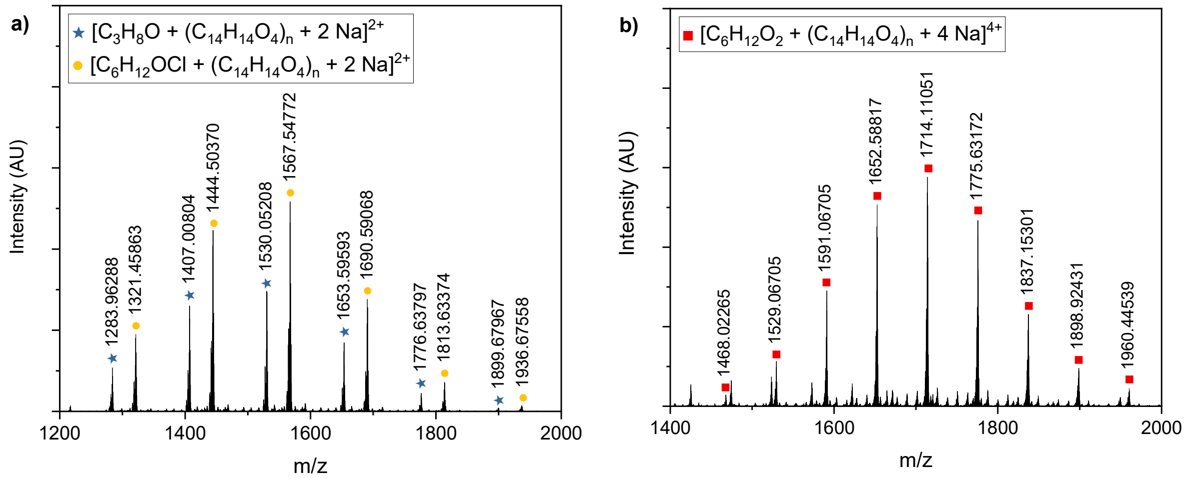

Figure S17. Average mass spectra from GPC-SEC-MS of polyester (PA/CHO) catalyzed by **1**/[PPN]Cl. Retention time range: a) 14.2–14.6 min, b) 12.8–13.0 min; positive ion mode.

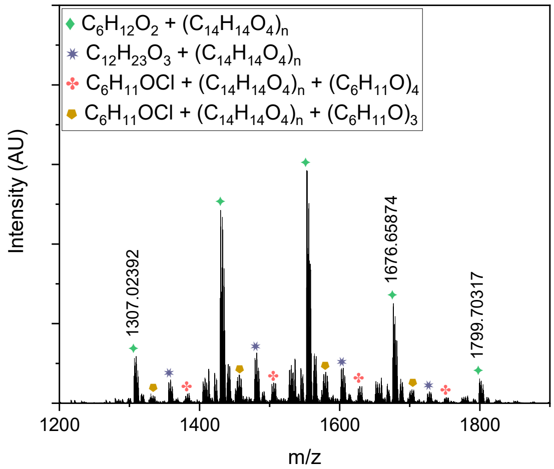

Figure S18. Average mass spectrum from GPC-SEC-MS of polyester (PA/CHO) catalyzed by **1**/[PPN]Cl in presence of 10 equiv. of *trans*-cyclohexane-1,2-diol. Retention time range: 14.2–14.6 min; positive ion mode. Identified signals are doubly charged z = 2 adducts of polymer with a combination Na^+, K^+, H_4N^+ and H^+ (only a single adduct is marked on the mass spectrum).

Table S5. Selected Examples of Catalysts for CHO/PA Copolymerization.

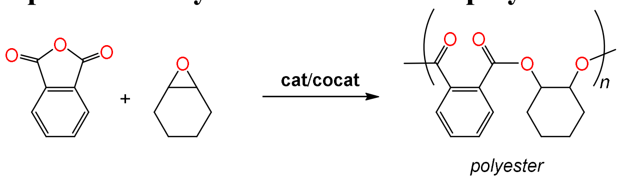

Entry	Complex/catalyst	Cocat.	Ratio Cat:CHO:PA	Temp. (°C)	TOF (h^{-1})
Monometallic system					
1	(salophen)CrCl[13] **1-Cr**	[PPN]Cl	1:250:250	110	245
2	(diamino-bisphenolate)CrCl[14] **2-Cr**	[PPN]Cl	1:800:100	100	50
3	(aminotriphenolate)Cr[15] **3-Cr**	DMAP	1:1600:800	100	380
4	(aminotriphenolate)TiCl[16] **4-Ti**	[PPN]Cl	1:250:250	110	124
5	$Zn(C_6F_5)_2$[17] **5-Zn**	DMAP	1:500:100	110	116
Bifunctional system					
6	$[(9\text{-BBN})B(CH_2)_5N(Me)_3]Cl$[18] **6-B**	-	1:400:200	120	258
Bimetallic system					
7	(*o*-vanillin)Di-Zn[19] **7-Zn$_2$**	-	1:800:100	100	198
8	(aminotriphenolate)Di-Fe[20] **8-Fe$_2$**	[PPN]Cl	1:2500:500	100	1180
9	(μ-biphenol)Di-Al[12] **9-Al$_2$**	[PPN]Cl	1:1000:250	50	750
10	(salophen)Di-Cr[21] **10-Cr$_2$**	[PPN]Cl	1:2000:250	60	912
11	(*o*-vanillin)Al(III)/K(I)[22] **11-Al/K**	-	1:2000:400	100	1072
12	(*o*-vanillin)Al(III)/Rb(I)[22] **11-Al/Rb**	-	1:2000:400	100	1136
13	(*o*-vanillin)Fe(III)/K(I)[23] **12-Fe/K**	-	1:2000:400	100	1152
14	(*o*-vanillin-C_2Me_2)[24] **13-Al/K**[a]	-	1:2000:400	100	1890

[a] Addition of 400 equiv. of *trans*-cyclohexane-1,2-diol.

Scheme S1. Structures of the Complexes Copolymerizing CHO/PA (Table S5).

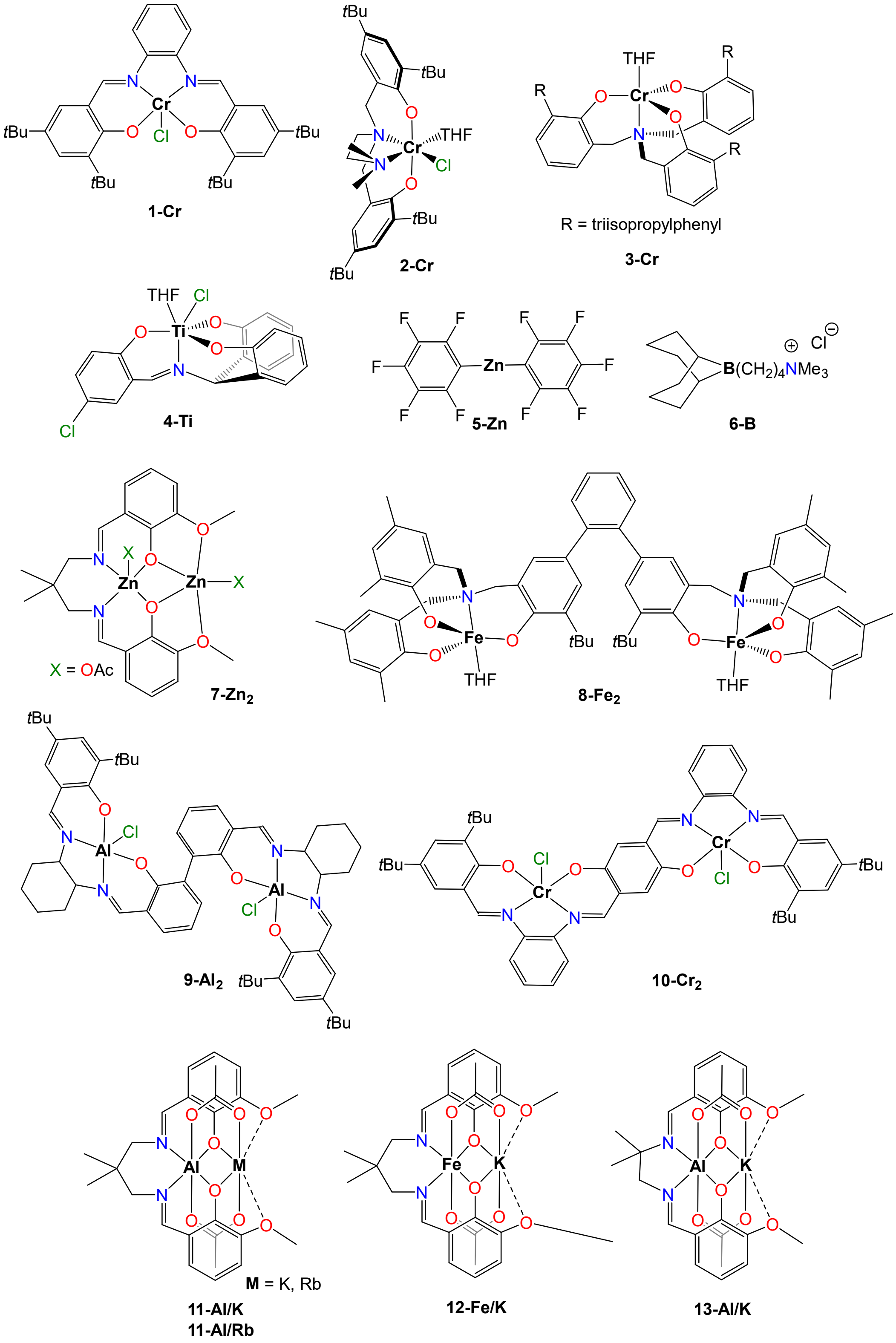

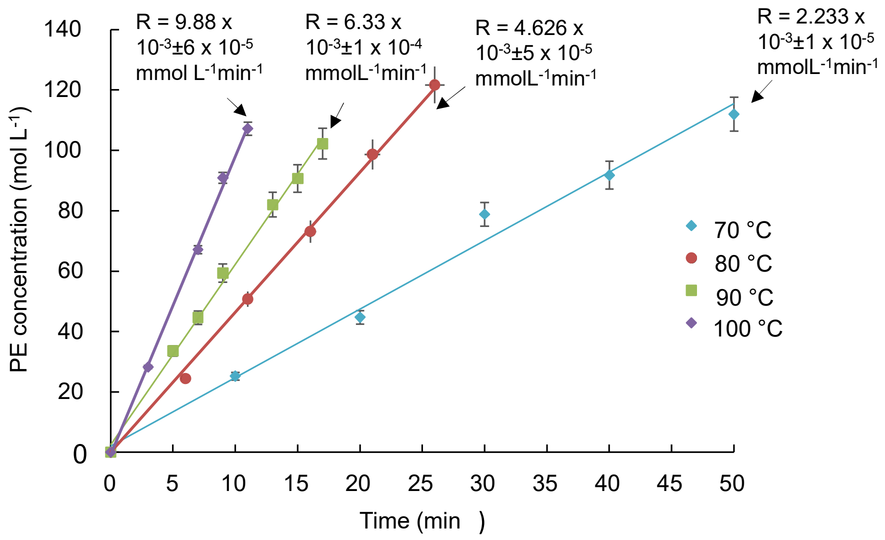

Figure S19. Overlay of polyester concentration vs. time plots for ROCOP of CHO and PA catalyzed by **1**/[PPN]Cl over temperatures 70 °C-100 °C. Average values of PE concentrations from multiple runs plotted with error $\pm\sigma/\sqrt{n}$. R = rate of reaction.

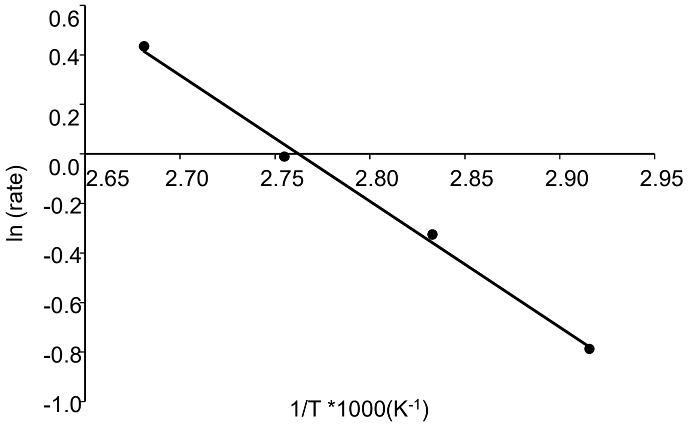

Figure S20. Arrhenius plot for ROCOP of PA and CHO catalyzed by **1**/[PPN]Cl. Y = -5.09+14.06, $R^2 = 0.99$.

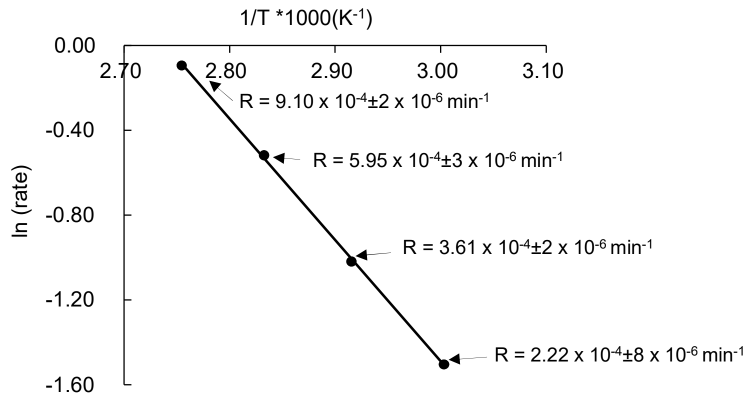

Figure S21. Arrhenius plot for ROCOP of PA and CHO catalyzed by **3**/[PPN]Cl. Y = -5.75+15.78, R^2 = 0.99. Average values of PE concentrations from multiple runs plotted with error $\pm\sigma/\sqrt{n}$. R = rate of reaction.

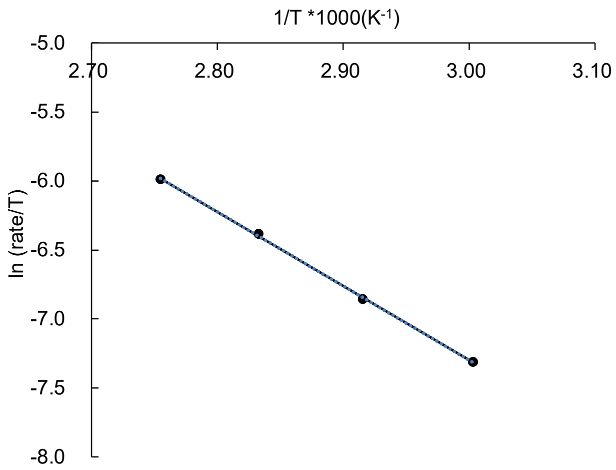

Figure S22. Eyring's plot for ROCOP of PA and CHO catalyzed by **3**/[PPN]Cl. Y = 5.40+8.91, R^2 = 0.99.

References:

(1) Fulmer, G. R.; Miller, A. J. M.; Sherden, N. H.; Gottlieb, H. E.; Nudelman, A.; Stoltz, B. M.; Bercaw, J. E.; Goldberg, K. I., NMR Chemical Shifts of Trace Impurities: Common Laboratory Solvents, Organics, and Gases in Deuterated Solvents Relevant to the Organometallic Chemist. *Organometallics* **2010,** *29*, 2176-2179.

(2) APEX2. Version 2014.11-0; Bruker-AXS, Inc., Madison, WI, USA, 2014.

(3) SAINT. Version 7.68A; Bruker-AXS, Inc., Madison, WI, USA, 2010

(4) Krause, L.; Herbst-Irmer, R.; Sheldrick, G. M.; Stalke, D., Comparison of Silver and Molybdenum Microfocus X-ray Sources for Single-crystal Structure Determination. *J. Appl. Crystallogr.* **2015,** *48*, 3-10.

(5) Sheldrick, G. M., SHELXT - Integrated Space-group and Crystal-structure Determination. *Acta Crystallogr., Sect. A: Found. Adv.* **2015,** *71*, 3-8.

(6) Sheldrick, G. M., Crystal Structure Refinement with SHELXL. *Acta Crystallogr., Sect. C: Cryst. Struct. Chem.* **2015,** *71*, 3-8.

(7) Lalrempuia, R.; Underhaug, J.; Törnroos, K. W.; Le Roux, E., Anionic Hafnium Species: an Active Catalytic Intermediate for the Coupling of Epoxides with CO_2? *Chem. Commun.* **2019,** *55*, 7227-7230.

(8) Jeske, R. C.; Rowley, J. M.; Coates, G. W., Pre-Rate-Determining Selectivity in the Terpolymerization of Epoxides, Cyclic Anhydrides, and CO_2: A One-Step Route to Diblock Copolymers. *Angew. Chem. Int. Ed.* **2008,** *47*, 6041-6044.

(9) Darensbourg, D. J.; Poland, R. R.; Escobedo, C., Kinetic Studies of the Alternating Copolymerization of Cyclic Acid Anhydrides and Epoxides, and the Terpolymerization of Cyclic Acid Anhydrides, Epoxides, and CO_2 Catalyzed by (salen)Cr^{III}Cl. *Macromolecules* **2012,** *45*, 2242-2248.

(10) Han, B.; Liu, B.; Ding, H.; Duan, Z.; Wang, X.; Theato, P., CO_2-Tuned Sequential Synthesis of Stereoblock Copolymers Comprising a Stereoregularity-Adjustable Polyester Block and an Atactic CO_2-Based Polycarbonate Block. *Macromolecules* **2017,** *50*, 9207-9215.

(11) Liu, Y.; Xiao, M.; Wang, S.; Xia, L.; Hang, D.; Cui, G.; Meng, Y., Mechanism Studies of Terpolymerization of Phthalic Anhydride, Propylene Epoxide, and Carbon Dioxide Catalyzed by ZnGA. *RSC Adv.* **2014,** *4*, 9503-9508.

(12) Li, J.; Liu, Y.; Ren, W.-M.; Lu, X.-B., Asymmetric Alternating Copolymerization of Meso-epoxides and Cyclic Anhydrides: Efficient Access to Enantiopure Polyesters. *J. Am. Chem. Soc* **2016,** *138*, 11493-11496.

(13) Hosseini Nejad, E.; van Melis, C. G. W.; Vermeer, T. J.; Koning, C. E.; Duchateau, R., Alternating Ring-Opening Polymerization of Cyclohexene Oxide and Anhydrides: Effect of Catalyst, Cocatalyst, and Anhydride Structure. *Macromolecules* **2012,** *45*, 1770-1776.

(14) Vos, C. W.; Beament, J.; Kozak, C. M., Ring Opening Polymerization and Copolymerization for Polyester and Polycarbonate Formation by a Diamino-bis(phenolate) Chromium(III) catalyst. *Polym. Chem.* **2023,** *14*, 5083-5093.

(15) Ryu, H. K.; Bae, D. Y.; Lim, H.; Lee, E.; Son, K.-s., Ring-opening Copolymerization of Cyclic Epoxide and Anhydride Using a Five-coordinate Chromium Complex with a Sterically Demanding Amino Triphenolate Ligand. *Polym. Chem.* **2020,** *11*, 3756-3761.

(16) Jeong, Y.; Cho, M. K.; Seo, S.; Cho, H.; Son, K.-s.; Kim, H., Electronic Tuning of Iminotriphenolate Ligands for Titanium(IV)-catalyzed Ring-opening Copolymerization of Cyclic Anhydrides and Epoxides. *ChemCatChem* **2023,** *15*, e202201086.

(17) Ji, H.-Y.; Wang, B.; Pan, L.; Li, Y.-S., Lewis Pairs for Ring-Opening Alternating Copolymerization of Cyclic Anhydrides And Epoxides. *Green Chem.* **2018,** *20*, 641-648.

(18) Xie, R.; Zhang, Y.-Y.; Yang, G.-W.; Zhu, X.-F.; Li, B.; Wu, G.-P., Record Productivity and Unprecedented Molecular Weight for Ring-Opening Copolymerization of Epoxides and Cyclic Anhydrides Enabled by Organoboron Catalysts. *Angew. Chem. Int. Ed.* **2021,** *60*, 19253-19261.

(19) Thevenon, A.; Garden, J. A.; White, A. J. P.; Williams, C. K., Dinuclear Zinc Salen Catalysts for the Ring Opening Copolymerization of Epoxides and Carbon Dioxide or Anhydrides. *Inorg. Chem.* **2015,** *54*, 11906-11915.
(20) Shi, Z.; Jiang, Q.; Song, Z.; Wang, Z.; Gao, C., Dinuclear Iron(III) Complexes Bearing Phenylene-bridged Bis(amino triphenolate) Ligands as Catalysts for the Copolymerization of Cyclohexene Oxide with Carbon Dioxide or Phthalic Anhydride. *Polym. Chem.* **2018,** *9*, 4733-4743.
(21) Cui, L.; Ren, B.-H.; Lu, X.-B., Trinuclear Salphen–chromium(III)chloride Complexes as Catalysts for the Alternating Copolymerization of Epoxides and Cyclic Anhydrides. *J. Polym. Sci.* **2021,** *59*, 1821-1828.
(22) Diment, W. T.; Gregory, G. L.; Kerr, R. W. F.; Phanopoulos, A.; Buchard, A.; Williams, C. K., Catalytic Synergy Using Al(III) and Group 1 Metals to Accelerate Epoxide and Anhydride Ring-Opening Copolymerizations. *ACS Catal.* **2021,** *11*, 12532-12542.
(23) Diment, W. T.; Rosetto, G.; Ezaz-Nikpay, N.; Kerr, R. W. F.; Williams, C. K., A Highly Active, Thermally Robust Iron(III)/Potassium(I) Heterodinuclear Catalyst for Bio-derived Epoxide/Anhydride Ring-opening Copolymerizations. *Green Chem.* **2023,** *25*, 2262-2267.
(24) Shellard, E. J. K.; Diment, W. T.; Resendiz-Lara, D. A.; Fiorentini, F.; Gregory, G. L.; Williams, C. K., Al(III)/K(I) Heterodinuclear Polymerization Catalysts Showing Fast Rates and High Selectivity for Polyester Polyols. *ACS Catal.* **2024,** *14*, 1363-1374.
